# Prediction of suitable distribution areas for Alismatis Rhizoma based on the MaxEnt model and study on quality-environment correlation

**DOI:** 10.3389/fpls.2026.1723386

**Published:** 2026-01-30

**Authors:** Zimin Wang, Zongyi Zhao, Gaoting Yang, Haotian Zhang, Jialin Li, Zhiqiong Lan

**Affiliations:** 1 Key Laboratory of Standardization of Chinese Medicine (Chengdu University of Traditional Chinese Medicine), Ministry of Education, Chengdu, China; 2 Pharmacy Department, Leshan Hospital of Traditional Chinese Medicine, Leshan, China

**Keywords:** Alisma orientale (Sam.) Juzep., Alisma plantago-aquatica Linn., morphological characteristics, soil enzymeactivity, suitable distribution area, triterpenoid constituents

## Abstract

**Background:**

Climate change profoundly impacts the suitable habitats of medicinal plants, and environmental factors in their production areas further determine their quality. Alismatis Rhizoma (AR) is a commonly used Chinese herbal medicine in clinical practice, derived from the dried rhizomes of *Alisma plantago-aquatica* Linn. and *Alisma orientale* (Sam.) Juzep., with an annual production exceeding 10,000 tons. However, the current uncontrolled expansion of cultivation has led to variations in the quality of medicinal herbs.

**Purpose:**

Clearly define the suitable distribution areas for the two source species of AR, reveal the relationship between the cultivation environment and the quality of the mainstream cultivar *A. plantago-aquatica*, and provide scientific support for optimizing planting layouts, improving medicinal quality, and responding to climate change.

**Methods:**

We employed for the first time a MaxEnt model optimized using the Kuenm package to systematically predict the suitable habitat distribution and its shifts for the two source species of AR under current and future climate scenarios. utilizing high-performance liquid chromatography, we conducted correlation analyses between 43 environmental factors and 15 medicinal quality indicators for the primary cultivated variety (*A. plantago-aquatica*), establishing stepwise regression models.

**Results:**

Precipitation, altitude, and temperature are the key environmental factors influencing both the potential distribution and quality of AR. Currently, highly suitable habitats are mainly located in southern and eastern China (*A. plantago-aquatica*: Sichuan, Guangxi, and Guangdong provinces; *A. orientale*: Fujian, Jiangxi, Guangdong, and Hunan provinces). Under future climate scenarios, the range of suitable habitats will expand towards higher latitudes, with the migration range for *A. plantago-aquatica* being 26.12°N - 30.07°N and that for *A. orientale* being 29.95°N - 31.72°N. This study also newly identifies significant correlations between soil properties-total potassium, available phosphorus, available manganese, available molybdenum, and urease activity-and the color, size, and active constituents of AR.

**Conclusions:**

The study has clarified the suitable habitats for the two source species of AR and the environment-quality coupling relationships of its mainstream cultivars. The integrated ecological-cultivation-quality control strategy developed provides critical guidance for advancing the ecological agriculture and enhancing the quality of AR.

## 1 Introduction

From a plant ecology perspective, climate change profoundly influences the geographic distribution ranges and suitable habitats of medicinal plants by altering environmental conditions such as temperature and precipitation (Zhao et al., 2024; Mechergui et al., 2025; Xiang et al., 2025a). From a crop cultivation perspective, environmental factors in the production area directly regulate the growth and development processes of medicinal plants, thereby determining the formation and accumulation of their superior traits and active medicinal components (Ma et al., 2024; Jiang et al., 2025; Liu Z. et al., 2025). The integration of these two disciplines will collectively reveal the comprehensive impact of environmental factors on medicinal plants, from “distribution feasibility” to the “formation of medicinal quality”. This integrated approach provides scientific guidance for optimizing suitable cultivation regions and habitat management for Chinese medicinal herbs, ultimately advancing ecological cultivation practices.

Alismatis Rhizoma (AR, known as Zexie in Chinese), a major medicinal material used in clinical traditional Chinese medicine, is widely utilized in East Asian regions, including China, Japan, and Korea (China Pharmacopoeia Commission, 2020; Japanese Pharmacopoeia Commission, 2021). It has a medicinal history of nearly 2,000 years and a cultivation history of over 500 years (Huang, 2006). After long-term cultivation and domestication, the Chinese medicinal material AR has diverged into two distinct botanical species: *Alisma plantago-aquatica* Linn. and *Alisma orientale* (Sam.) Juzep., which are cultivated in different regions within China and exhibit distinct quality characteristics. Historically, the major production areas of AR in China have undergone significant geographical shifts: commencing in the Henan around the beginning of the Common Era, later shifting to Shaanxi; expanding to Fujian during the Ming and Qing dynasties, where it became an authentic producing area; and further extending to Sichuan and Jiangxi during the Republican period (Peng et al., 2013; Wang et al., 2022). As the primary producer, China’s annual production and sales volume of AR exceeds 10,000 tons, with substantial exports to Japan, South Korea, and other countries (Wen, 2021). In recent years, the cultivation pattern of AR has undergone tremendous changes. Concurrent with a sharp decline in the cultivation of *A. orientale* in its primary regions of Fujian and Jiangxi, *A. plantago-aquatica* has been extensively cultivated in Sichuan and surrounding areas, now accounting for over 90% of the total output and giving rise to many new production regions beyond traditional bases like Leshan and Meishan. However, the quality of AR in the market is inconsistent and varies significantly (Cai et al., 2016). Therefore, to scientifically plan the production areas for AR, enhance the production efficiency of the cultivation industry, and ensure and improve the quality of these materials, it is imperative to research the ecological suitability of AR and the environmental factors affecting the quality of its medicinal materials.

However, regarding ecological suitability research on AR, only one study by Lin et al. (2011) has been reported, utilizing the “Geographical Information System for Suitable Area Analysis of Traditional Chinese Medicinal Materials” (TCMGIS) to explore suitable distribution regions for *A. orientale*. In contrast, no related studies have been published on *A. plantago-aquatica*, which is currently the dominant species accounting for more than 90% of both production and sales. Compared with the TCMGIS model, the Kuenm-optimized MaxEnt model adopted in this study demonstrates distinct advantages in three aspects. First, in terms of prediction accuracy, it can adaptively optimize and characterize the nonlinear relationships among environmental factors, thereby mitigating overfitting (Van Steenderen and Sutton, 2024). Second, regarding parameter stability, it adopts delta. AICc minimization as the objective calibration criterion, which enables the omission rate to be stably controlled within 0.03 (Cobos et al., 2019). Third, in terms of multi-species adaptability, it is compatible with both continuous and categorical variables, and can effectively characterize niche differentiation across different species within a unified framework. Therefore, this model exhibits strong universality for both widespread and endemic species (Shen et al., 2024; Gao et al., 2025). In conclusion, this parameterized and optimizable framework provides a more flexible and robust forecasting solution.

Regarding the influence of climatic factors on AR quality, Chu et al. (2007) indicated that the average relative humidity of the production area was positively correlated with the content of 23-Acetyl alisol B (23-AAB) in the medicinal material of *A. orientale*. In contrast, Zhang et al. (2009) indicated that the altitude, sunshine duration, annual precipitation, annual average temperature, and the maximum or minimum temperatures of the cultivated areas in Sichuan had no significant correlation with the contents of 23-AAB and 24-Acetyl alisol A (24-AAA) in *A. orientale.* More recently, Lang et al. (2023) discovered that under effective accumulated temperature conditions, both *A. plantago-aquatica* (Pengshan, Sichuan) and *A. orientale* (Jian’ou, Fujian) exhibited high levels of 23-AAB but low levels of 23-Acetyl alisol C (23-AAC). Overall, conclusions regarding the correlation between climatic factors and the triterpenoid content in AR vary among different scholars. These discrepancies may stem from the fluctuating climatic conditions across different years, the timing of sample collection, or the combined effects of environmental factors such as soil characteristics in different production areas.

Regarding soil factors affecting AR quality, Chu et al. (2007) reported that soil potassium (K) had the greatest impact on the 23-AAB content in *A. orientale* compared to the other 11 trace elements analyzed. Yang et al. (2012b) found no significant correlation in Pengshan, Sichuan soils between the content of the triterpenoid 23-AAB in AR and the levels of total nitrogen (TN), total phosphorus (TP), total potassium (TK), alkali-hydrolyzable nitrogen (AN), available phosphorus (AP), or available potassium (AK). However, TN and TK showed a highly significant positive correlation with the alcohol-soluble extract content of the medicinal materials. Yang et al. (2024a) reported that among 13 mineral elements in soils from 2 A*. plantago-aquatica* production areas (Dongpo District and Pengshan County, Sichuan Province), K, Cu, and Zn significantly influenced the accumulation of 6 terpenoid compounds in the medicinal material. Subsequently, Yang (2025) further discovered that in four production areas (Dongpo District and Pengshan County in Meishan City; Jiajiang County and Wutongqiao District in Leshan City, Sichuan), soil pH, total nitrogen (TN), total manganese (Mn), and total zinc (Zn) were significantly or highly significantly positively correlated with the overall content of 6 triterpenoid monomers in *A. plantago-aquatica*. However, the correlations between specific soil physicochemical indicators and the contents of individual triterpenoid active components were inconsistent. Although *A. plantago-aquatica* is an aquatic plant, modern research indicates that the contents of its trace elements and triterpenoid active components are not directly related to water quality, as the influence of water quality on the medicinal material is exerted indirectly through the soil medium (Wen, 2021; Wu et al., 2004).

It is noteworthy that previous studies on the relationship between the quality of AR and environmental factors have produced inconsistent and even contradictory conclusions. For example, early research (Yang et al., 2012b) reported no significant correlation between TN and 23-AAB content, whereas recent studies (Yang, 2025) indicate an association between the two. This inconsistency highlights that the formation of medicinal herb quality is the result of the combined effects of multiple environmental factors (Liu et al., 2023; Huang et al., 2025). Previous research has suffered from three primary limitations. Firstly, the selection of environmental factors, particularly concerning soil, has been narrow, predominantly focusing on physicochemical properties and inorganic elements, while lacking a comprehensive evaluation of key biochemical indicators such as bioavailable elements and enzyme activities. Secondly, the quality assessment of AR has been overly reliant on a few triterpenoid monomers, failing to integrate multidimensional indicators like macroscopic morphology, extractables, and total triterpenoid content, which are crucial for systematically evaluating its overall quality. Thirdly, past research has primarily focused on species *A. orientale*, with insufficient attention given to the dominant species *A. plantago-aquatica*, thereby failing to guide the scientific planning of cultivation sites for AR. In summary, although exploratory research on the suitable habitats and quality-environment relationships of AR has provided a foundation and yielded some progress, more systematic studies are imperative to genuinely guide the scientific planning and cultivation practices for this Chinese medicinal herb.

In terms of research methodologies, the researchers employed multiple modeling approaches to assess the ecological suitability of medicinal plants. Widely recognized models include Generalized Linear Model (GLM), the BIOCLIM bioclimatic analysis system model, Artificial Neural Networks, Random Forest (RF), and Maximum Entropy Model (MaxEnt) (Elith and Leathwick, 2009; Guo et al., 2020). Among these, the MaxEnt model has demonstrated significant advantages for predicting species’ potential suitable distributions owing to its high predictive accuracy, broad applicability, superior computational efficiency, relatively straightforward implementation, and fast processing speed. It is capable not only of effectively predicting suitable habitat ranges and shifts under climate change but also of quantifying the contributions of climatic factors (Zhang H. et al., 2024; Liu Q.W et al., 2025; Xiang et al., 2025b). These strengths have established MaxEnt as the modeling tool of choice (Phillips et al., 2006; Elith and Leathwick, 2009; Merow et al., 2013; Fletcher et al., 2019). In recent years, it has been increasingly applied to analyze the relationships between medicinal plant distribution and environmental variables, such as predicting potential suitable habitats for medicinal plants like *Leonurus japonicus* Houtt., *Meconopsis aculeata*, African wormwood, *Bletilla striata* (Thunb.) Rchb.f., *Panax notoginseng*, *Paris polyphylla* var. *Yunnanensis* (Chen et al., 2024; Paul and Samant, 2024; Matsane et al., 2025; Mu et al., 2025, Wu et al., 2025a, Zhong et al., 2025) under climate change scenarios. Furthermore, recent research has shown that the Kuenm package in R provides a more rigorous model optimization process, automatically selecting optimal MaxEnt modeling parameters (Cobos et al., 2019): the regularization multiplier (RM) and Feature combination (FC). This enhances the accuracy and stability of MaxEnt-constructed niche models. However, to date, no studies have been reported that apply the MaxEnt model to determine the suitable distribution range of AR.

**Figure 1:**
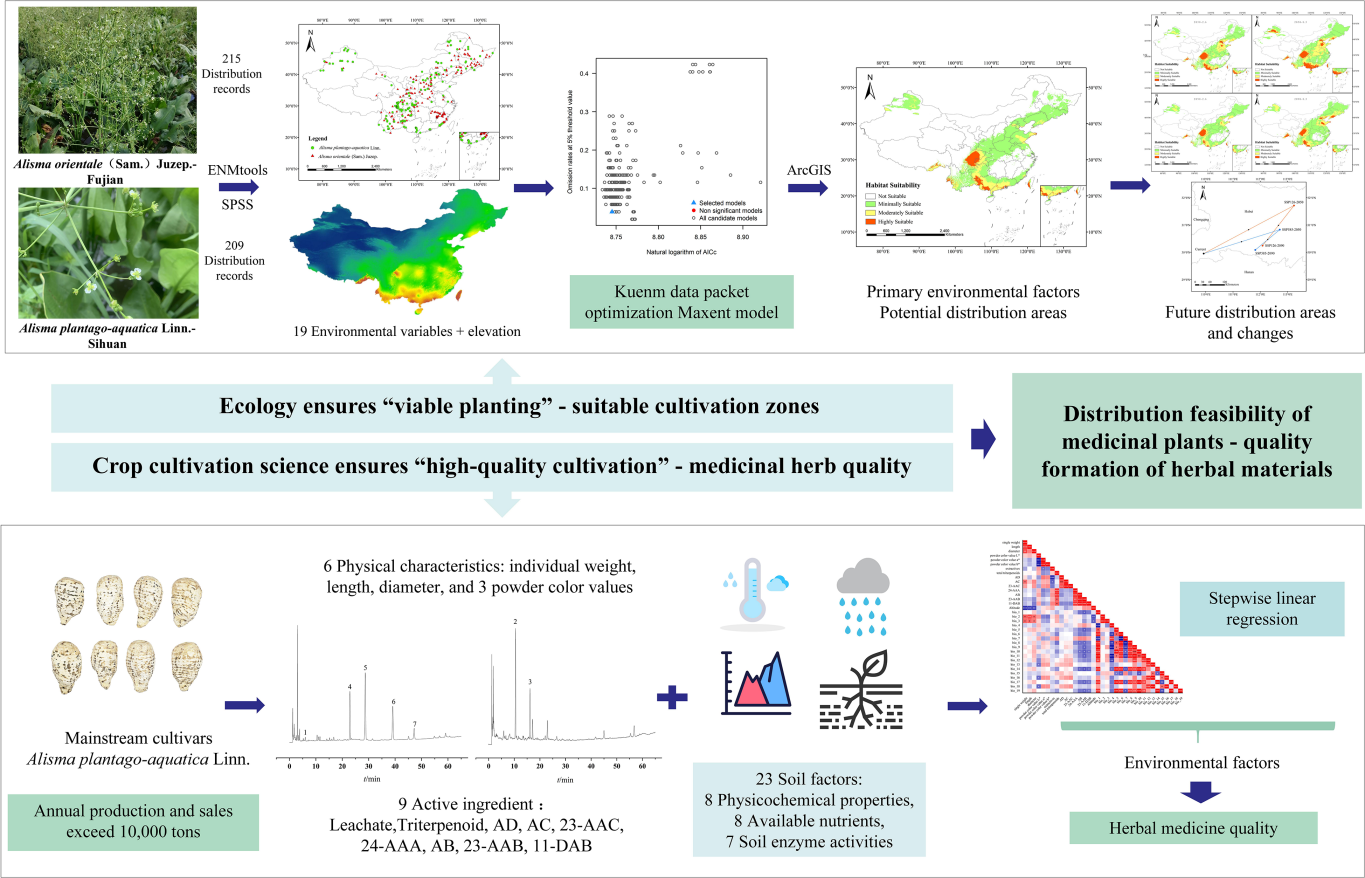
Schematic diagram of the predicted suitable distribution areas for AR and the research approach for quality-environment correlation studies.

This study applies the MaxEnt model, widely utilized in species ecological suitability assessment, to analyze the ecological suitability of AR. Using the Kuenm package for parameter optimization, we identify suitable distribution regions for the two source plants of AR: *A. plantago-aquatica* and *A. orientale*. By visual analysis with ArcGIS software, we predict the suitable areas for both species under current and future climate scenarios (2050s and 2090s), analyze their spatial variation and expansion trends, and provide a scientific basis for regional planning of AR medicinal production. Furthermore, building upon prior research, this study focuses on the mainstream cultivar *A. plantago-aquatica* in modern AR cultivation. Through correlation and stepwise regression analyses, we comprehensively assess the relationships and synergistic effects of 43 environmental factors (including climate and soil variables) on 15 quality indicators of AR, encompassing both morphological traits and active constituents. For the first time, a bidirectional multidimensional indicator system is employed to identify key environmental factors influencing AR quality. Compared to prior studies, this research innovatively integrates multidisciplinary approaches-combining plant ecology, crop cultivation science, and the traditional Chinese medicine concept of “quality evaluation through morphological identification” with modern analytical techniques such as Geographic Information Systems (GIS) and high-performance liquid chromatography (HPLC). This integrated methodology yields more targeted, systematic, and forward-looking insights, offering a scientific foundation for optimizing AR cultivation zones. Consequently, it supports improved efficiency in AR cultivation and enhances both the yield and quality of medicinal materials. The overall research approach and strategy for this paper are illustrated in **Figure 1**.

## 2 Materials and methods

### 2.1 Acquisition and processing of species distribution data and environmental variables

**Figure 2:**
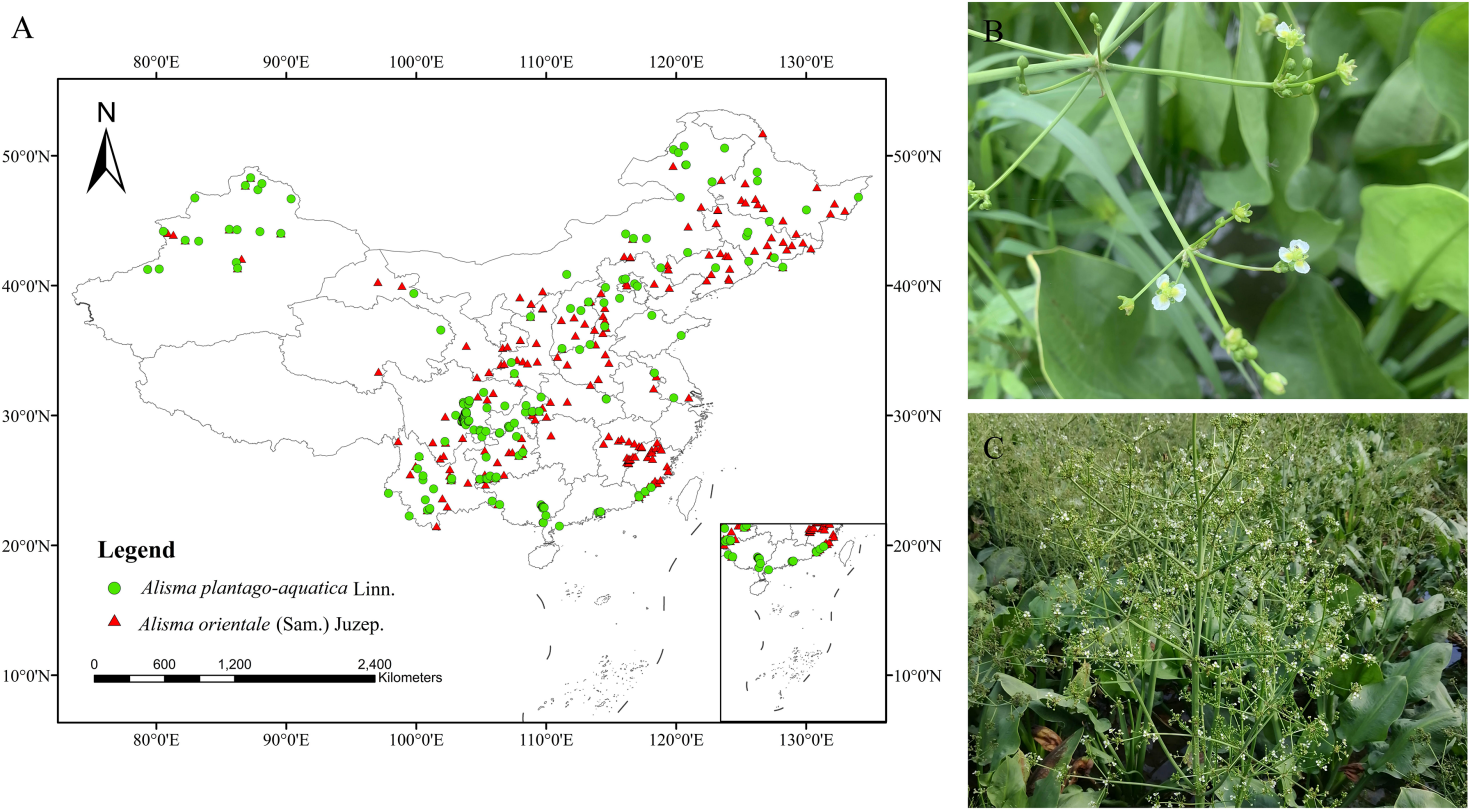
**(A)** Spatial distribution of *A*. *plantago-aquatica* and *A*. *orientale.***(B)***A*. *plantago-aquatica* (Sichuan). **(C)***A*. *orientale* (Fujian).

The species distribution records of *A. plantago-aquatica* and *A. orientale* were obtained from databases (GBIF, https://www.gbif.org/; NSII, http://www.nsii.org.cn/; CVH, https://www.cvh.ac.cn/), relevant literature (Zhang et al., 2010; Yang, 2012a; Dai et al., 2020; Li et al., 2020), and preliminary survey data collected by the research team. For locations lacking latitude and longitude coordinates, the Baidu Map Coordinate Picking System was used to supplement the missing coordinates, while incorrect and duplicate distribution points were removed. Given the 30″ resolution of the climate data, to minimize errors arising from cluster distribution characteristics (Li et al., 2016; Zhao et al., 2024) and avoid spatial autocorrelation, we retained only one distribution point per 1 km × 1 km grid cell using the ENMtools toolbox (Warren et al., 2010). Ultimately, 209 distribution records for *A. plantago-aquatica* and 215 distribution records for *A. orientale* were obtained. The data were saved in CSV format for building the MaxEnt model (see **Figure 2** for species distribution maps). Additionally, all fundamental geographic information data used in this study were obtained from the National Geographic Information Public Service Platform (https://www.tianditu.gov.cn/), with map review approval number GS (2024) 0650. The base map remained unmodified, and all spatial data were processed using ArcGIS 10.8.2 software.

Current climate data (19 bioclimatic variables and elevation, **Table 1**) and future climate data (for the periods 2041–2060 and 2081-2100) were sourced from the WorldClim global climate database (Version 2.1) and the BCC-CSM2-MR model of CMIP6, respectively. Future climate scenarios include two representative pathways: SSP1-2.6 (low radiative forcing) and SSP5-8.5 (high radiative forcing), assuming stable topography. All environmental variables had a spatial resolution of 30 seconds, were projected in the WGS84 coordinate system, and were converted to ASCII format compatible with MaxEnt. To avoid overfitting due to multicollinearity, an initial preliminary model run was conducted using MaxEnt (version 3.4.0) to assess the contribution rate of each variable, and the values of each variable at species occurrence points were extracted using ArcGIS. Subsequently, Pearson correlation coefficients (|r|) between variables were calculated using SPSS 26.0. The final criteria for variable selection were |r| < 0.85 (Li et al., 2024; Zhang Y. et al., 2024), with priority given to variables showing higher contribution rates in the preliminary simulation. These selected variables were used for subsequent model construction and optimization. The final selected variable contribution values are shown in **Supplementary Table S1**.

**Table 1:** Bioclimatic variable information.

Abbreviation	Ecological factors	Units
bio1	Annual mean temperature	°C
bio2	Mean diurnal temperature range	°C
bio3	Isothermality	–
bio4	Temperature seasonality	–
bio5	Max temperature of warmest month	°C
bio6	Min temperature of coldest month	°C
bio7	Temperature annual range	°C
bio8	Mean temperature of wettest quarter	°C
bio9	Mean temperature of driest quarter	°C
bio10	Mean temperature of warmest quarter	°C
bio11	Mean temperature of coldest quarter	°C
bio12	Annual precipitation	mm
bio13	Precipitation of wettest month	mm
bio14	Precipitation of driest month	mm
bio15	Precipitation seasonality	–
bio16	Precipitation of wettest quarter	mm
bio17	Precipitation of driest quarter	mm
bio18	Precipitation of warmest quarter	mm
bio19	Precipitation of coldest quarter	mm
altitude	Altitude	m

### 2.2 Modeling species distribution

The processed species distribution data and selected environmental variables were imported into the MaxEnt model. The model was configured as follows: 75% of the occurrence records were used for training and 25% for testing; the number of background points was set to 10,000; the random seed option was enabled; the model was run with 10 replicates; the output format was set to logistic; and all other parameters were kept at their default values. The final output represents the average of the 10 replicate runs (Elith et al., 2006). The predictive performance of the MaxEnt models is mainly affected by the RM and FC parameters. In this study, model parameters were optimized using the Kuenm package in R version 3.6.3. The tested parameter ranges included RM values from 0 to 4 (with a step size of 0.5, resulting in 8 levels) and 29 FC combinations comprising Linear (L), Quadratic (Q), Product (P), Threshold (T), and Hinge (H) features. A total of 232 candidate models (29 FCs × 8 RMs) were evaluated. Model complexity was determined based on a 5% training omission rate and delta AICc values. The model with a delta AICc value of 0 was selected as the optimal model (Cobos et al., 2019).

Model predictive accuracy was evaluated using the area under the receiver operating characteristic curve (ROC). The values range from 0 to 1, with higher values indicating a better model fit. The specific classification criteria were as follows: AUC < 0.6 (failure), 0.6-0.7 (poor), 0.7-0.8 (fair), 0.8-0.9 (good), > 0.9 (excellent) (Swets, 1988). Based on the habitat suitability values (0-1) derived from model outputs, the Jenks natural breakpoint method (Chen et al., 2013) was employed to classify the potential distribution of the study species into four habitat suitability categories: unsuitable (< 0.2), low suitability (0.2-0.4), moderate suitability (0.4-0.6), and high suitability (0.6-1). To ensure comparability between current and future climate scenarios, the same threshold was applied for classification in both cases.

### 2.3 AR and soil sample collection

In the current AR medicinal herb cultivation industry, *A. plantago-aquatica* serves as the mainstream cultivar, extensively cultivated especially in Sichuan and surrounding regions. Its production accounts for over 90% of the nation’s total AR output, with new production areas continuing to expand. However, previous research has predominantly focused on species *A. orientale*, while attention to the actual primary cultivated variety *A. plantago-aquatica* has been relatively limited. Therefore, this study aims to conduct a systematic quality evaluation and analysis of species *A. plantago-aquatica* to address this research gap in the field. Fresh rhizomes and soil samples of AR were collected from January to February 2025 in Sichuan, Guangxi, and Chongqing, China’s primary cultivation regions for this plant. These areas include traditional production zones such as Meishan and Leshan in Sichuan, as well as Guigang in Guangxi, alongside newer production areas like Mianyang and Deyang in Sichuan and Chongqing. The plant material was identified by Associate Professor Lan Zhiqiong of Chengdu University of Traditional Chinese Medicine as *A. plantago-aquatica* of the Alismataceae family of plants. Processing AR fresh tubers into AR medicinal materials combines the Chinese Pharmacopoeia with actual local processing methods, specifically: Fresh tubers are washed, air-dried at room temperature for 24 hours, hot-air dried at 50°C for 48 hours, then left at room temperature for 24 hours. They are dried again at 50°C until the moisture content falls below 14.0%. Subsequently, fibrous roots and coarse skin are removed, and the material is pulverized and sieved (using a No. 4 sieve) for later use. Soil samples were collected using the “S”-shaped multi-point sampling method from the root zone (depth 2–20 cm) around plant roots. Samples were thoroughly mixed, then divided using the quartering method. After thorough mixing, samples were subdivided by quartering, air-dried, ground, sieved, and stored for subsequent analysis. Based on the primary production areas of AR, we selected these 14 sampling sites, detailed sample collection information is provided in **Table 2**.

**Table 2:** Sample information collection form.

Sample no.	Location	Longitude (E)	Latitude (N)	Elevation(m)	Medicinal part	Soil and type	Vegetation type
Z1	Pengshan, Sichuan	103°48’28″	30°13’31″	393	tuber	Soil for growth/Paddy soil	Agricultural vegetation
Z2	Dongpo, Sichuan	103°44’3″	29°59’2″	383	tuber	Soil for growth/Paddy soil	Agricultural vegetation
Z3	Wutongqiao, Sichuan	103°40’40″	29°26’44″	343	tuber	Soil for growth/Paddy soil	Agricultural vegetation
Z4	Jiajing, Sichuan	103°36’13″	29°46’49″	371	tuber	Soil for growth/Paddy soil	Agricultural vegetation
Z5	Shizhong, Sichuan	103°47’19″	29°37’37″	350	tuber	Soil for growth/Paddy soil	Agricultural vegetation
Z6	Jingyan, Sichuan	104°1’26″	29°35’48″	344	tuber	Soil for growth/Paddy soil	Agricultural vegetation
Z7	Qianwei, Sichaun	103°58’1″	29°25’49″	383	tuber	Soil for growth/Paddy soil	Agricultural vegetation
Z8	Emeishan, Sichuan	103°33’28″	29°32’46″	419	tuber	Soil for growth/Paddy soil	Agricultural vegetation
Z9	Xingwen, Sichaun	105°3’7″	28°20’27″	409	tuber	Soil for growth/Paddy soil	Agricultural vegetation
Z10	Zitong, Sichuan	105°8’39″	31°36’5″	443	tuber	Soil for growth/Paddy soil	Agricultural vegetation
Z11	Jingyang, Sichuan	104°20’20″	31°12’12″	547	tuber	Soil for growth/Paddy soil	Agricultural vegetation
Z12	Dianjiang, Chongqing	107°29’38″	30°20’29″	415	tuber	Soil for growth/Paddy soil	Agricultural vegetation
Z13	Gangnan, Guangxi	109°48’33″	22°59’23″	50	tuber	Soil for growth/Paddy soil	Agricultural vegetation
Z14	Gangnan, Guangxi	109°45’17″	22°58’15″	53	tuber	Soil for growth/Paddy soil	Agricultural vegetation

### 2.4 Instruments and reagents

The high-speed centrifuge used in this study was provided by Shanghai Anting Scientific Instruments Co., Ltd. (Shanghai, China). The analytical balance with 0.1 mg accuracy (model BSA224S) was supplied by Sartorius AG (Göttingen, Germany). The spectrocolorimeter (model CM-5) was provided by Konica Minolta Holdings, Inc. (Tokyo, Japan). The pH meter (model FE28) was supplied by Mettler-Toledo LLC (Columbus, OH, USA). The flame photometer (model FP-6450) was provided by Shanghai Xinyi Microwave Chemical Technology Co., Ltd. (Shanghai, China). The inductively coupled plasma optical emission spectrometer (iCAP 7200 HS Duo), inductively coupled plasma mass spectrometer (iCAP RQ), and high-performance liquid chromatography system (Thermo Ultimate 3000) were all supplied by Thermo Fisher Scientific Inc. (Waltham, MA, USA). The digestion system (model SPH120) and Kjeldahl nitrogen analyzer (model KN-520) were provided by Alfa Laval Technology Co., Ltd. (Shanghai, China). The full-wavelength microplate reader (model SpectraMax 190) was supplied by Molecular Devices, LLC (San Jose, CA, USA). The digital bottle-top titrator (model WF08-Titrette) was provided by BRAND GmbH + Co KG (Wertheim, Germany).

Reference standards (all with purity > 98.0%) include alismoxide (AD, MUST-21081709), Alisol C (AC, MUST-24122511), 23-AAC (MUST-24030321), 24-AAA (MUST-23030914), Alisol B (AB, MUST-24060414), 23-AAB (MUST-24061301), and 11-Deoxy alisol B (11-DAB, MUST-24101911), all supplied by Chengdu Manster Biotechnology Co., Ltd. (Sichuan, China). Acetonitrile (HPLC grade) was supplied by Thermo Fisher Scientific Inc. (Fair Lawn, New Jersey, USA). All other chemical reagents were of analytical grade, also supplied by Chengdu Manster Biotechnology Co., Ltd. (Sichuan, China). Ultrapure water was supplied by Hangzhou Wahaha Group Co., Ltd. (Hangzhou, China).

### 2.5 Quantitative assessment of external characteristics of AR

According to the theory of “quality evaluation through morphological identification” in traditional Chinese medicine, and with reference to the 1984 “Seventy-six Kinds Of Medicinal Materials Commodity Specification Standards” and the 2018 “Commercial grades for Chinese materia medica- Alismatis Rhizoma”, AR is considered to be of high quality when it is large in size and white in color (National Medical Products Administration, 1984; China Association of Traditional Chinese Medicine, 2018). Previous research by our group also found that the individual weight and length of AR are significantly correlated with the content of its active triterpenoid components (Zhao et al., 2023). Therefore, this study objectively measured the size and color of AR based on its individual weight, length, diameter, and powder color values L^*^, a^*^, and b^*^ (where L^*^ represents lightness, a^*^ represents the red-green direction, and b^*^ represents the yellow-blue direction) to scientifically evaluate the appearance quality of the herbal material.

### 2.6 Determination of pharmacologically active components in AR

Modern research has demonstrated that triterpenoids constitute the primary bioactive constituents of AR, including 23-AAB, 23-AAC, AD, AC, 24-AAA, AB, and 11-DAB. Among these, the AB content in Sichuan-produced AR was significantly higher than that in Fujian-produced AR (approximately 6 times higher) (Zhang C. et al., 2023). AB serves as a potential quality marker for this medicinal material (Zhang H. et al., 2019). Therefore, building upon the chemical indicators for total extractables and the sum of 23-AAB and 23-AAC stipulated in the 2025 edition of the Chinese Pharmacopoeia, this study introduced additional measurements for total triterpenoids, AD, AC, 24-AAA, AB, and 11-DAB. For the first time, a comprehensive evaluation system for AR herbal materials was established, encompassing nine bioactive component indicators.

**Figure 3:**
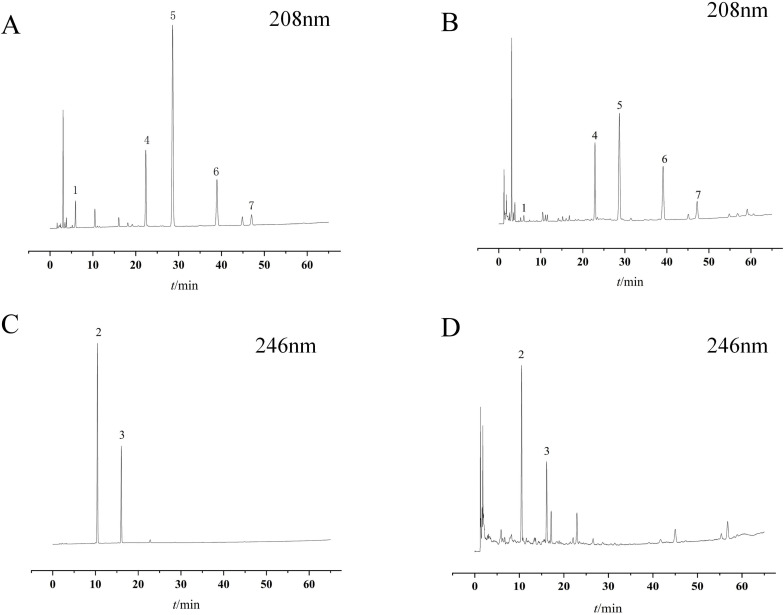
Chromatograms of reference standards and samples. **(A, C)** Reference standards. **(B, D)** Samples. 1. AD. 2. AC. 3. 23-AAC. 4. 24-AAA. 5. AB. 6. 23-AAB. 7. 11-DAB.

This study utilized an Agilent ZORBAX SB-C18 column (250 mm × 4.6 mm, 5 μm) for chromatographic analysis. The conditions were as follows: Water (phase A) - acetonitrile (phase B). The gradient elution program for the mobile phase is: 0–15 min, 40%-65% B; 15–20 min, 65%-68% B; 20–30 min, 68%-70% B; 30–65 min, 70%-95% B; Flow rate: 1 mL/min; Column temperature: 30°C; Detection wavelengths: 208 nm and 246 nm; Injection volume: 20 μL. The HPLC chromatograms of the reference standard and test sample solutions are shown in **Figure 3**.

Follow these steps for sample preparation and method validation: Take approximately 1.0 g of powdered sample processed according to sections 2.3, weigh accurately, precisely add 25 mL of acetonitrile, and perform ultrasonic extraction (40 kHz, 250 W) for 30 minutes. After cooling, reweigh the sample and replenish the weight loss with acetonitrile. The resulting solution was filtered through a 0.45 μm microporous membrane filter and subjected to HPLC analysis. Reference standards were accurately weighed and dissolved in acetonitrile to prepare standard stock solutions with the following concentrations: AD, 13.0 μg/mL; AC, 89.7 μg/mL; 23-AAC, 55.9 μg/mL; 24-AAA, 149.5 μg/mL; AB, 187.2 μg/mL; 23-AB, 184.6 μg/mL; and 11-DAB, 18.2 μg/mL. A calibration curve was constructed using these solutions. The method was validated for stability (0, 2, 4, 6, 8, 12, and 24 h), precision, repeatability, and recovery rate. The results are presented in **Table 3**. Additionally, the extractable content was determined using the hot extraction method described in General Rule 2201 of the Pharmacopoeia of the People’s Republic of China (2020 Edition, Volume IV). The total triterpenoid content was quantified according to a previously established method by the research group (Lin et al., 2023), using 23-AAB as the reference standard.

**Table 3:** Linear regression equations and correlation coefficients.

Component	Regression equation	*R* ^2^	Regression equation(μg)	Repeatability RSD(%)	Stability RSD(%)	Precision RSD(%)	Sample recovery rate(%)
AD	*Y* = 0.4708 *X* - 0.0385	0.9996	0.5-13.0	1.11	2.70	2.78	95.30
AC	*Y* = 0.4159 *X* - 0.0239	0.9998	3.45-89.7	2.61	1.07	0.46	102.50
23-AAC	*Y* = 0.3269 *X* - 0.0627	0.9999	2.15-55.9	1.87	0.46	0.98	99.18
24-AAA	*Y* = 0.1873 *X* + 0.0402	0.9997	5.75-149.5	1.76	0.62	0.30	102.55
AB	*Y* = 0.2001 *X* + 0.0442	0.9999	7.2-187.2	0.93	0.71	0.89	102.47
23-AAB	*Y* = 0.2033 *X* + 0.0283	0.9999	7.1-184.6	0.65	0.40	0.16	103.59
11-DAB	*Y* = 0.334 *X* - 0.0245	0.9996	0.7-18.2	1.65	1.87	0.41	101.14

### 2.7 Soil factor determination

We employed the glass electrode method to determine pH content (NY/T 1121.2-2006). The potassium dichromate oxidation-external heating method to determine organic carbon (OM) content (NY/T 1121.6-2006). Sulfuric acid-accelerator digestion followed by the Kjeldahl method to determine total nitrogen (TN) content (LY/T 1228-2015). NaOH alkali fusion with molybdenum-antimony spectrophotometry for TP determination (GB/T 9837-1988). NaOH alkali fusion with flame photometry for TK determination (NY/T 87-1988). Alkali hydrolysis diffusion method for soil AN determination (LY/T 1228-2015). Determination of soil AP content by molybdenum-antimony colorimetric method (NY/T 1121.7-2014). Determination of AK content by ammonium acetate extraction-flame photometer method (NY/T 889-2004). DTPA-TEA extraction method for determining available iron, manganese, copper, and zinc content (HJ 804-2016). Hot water reflux extraction method for determining available boron content (NY/T 1121.8-2006). Oxalic acid-ammonium oxalate extraction followed by ICP-MS determination of available molybdenum content (NY/T 1121.9-2012). Ammonium acetate exchange method for determining exchangeable calcium and magnesium content (NY/T 1615-2008). In addition, reagent kits (Nanjing MoFan Biotechnology Co., Ltd.) were used to determine the activity of soil urease, acid phosphatase, neutral phosphatase, sucrase, polyphenol oxidase, neutral protease, and peroxidase.

## 3 Results and analysis

### 3.1 MaxEnt model optimization results and analysis of key environmental factors

**Figure 4:**
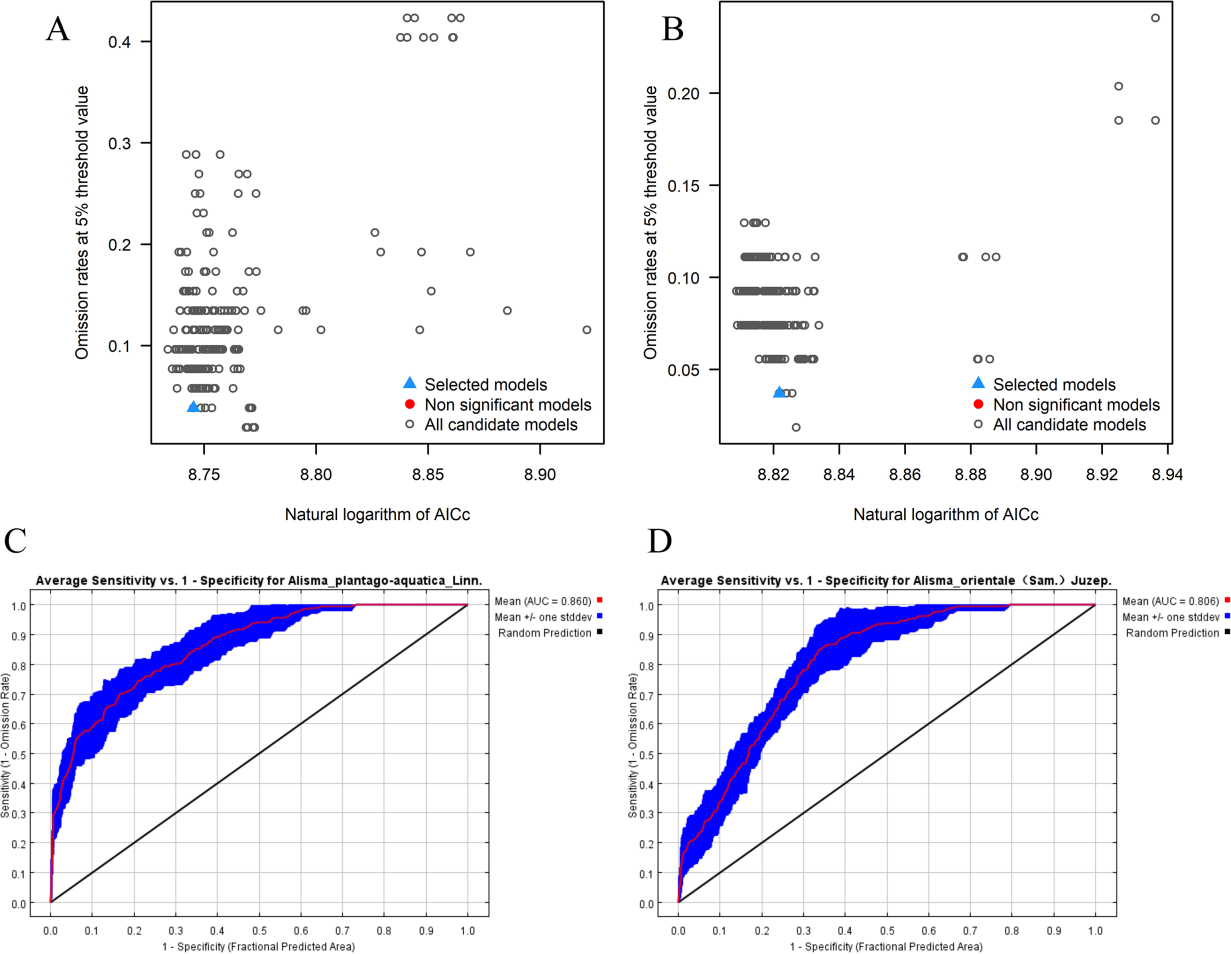
**(A, C)** Optimized results of the MaxEnt model for *A. plantago-aquatica* and ROC curves. **(B, D)** optimized results of the MaxEnt model for *A. orientale* and ROC curves.

Based on delta AICc = 0 and other evaluation metrics, the Kuenm software package was used to optimize the models. The optimal parameter configurations for *A. plantago-aquatica* and *A. orientale* were determined as FC = QPH, RM = 2, and FC = LH, RM = 4, respectively. Using this combination, a final distribution model was established for prediction. The average AUC values from 10 replicate runs of the MaxEnt model were 0.860 and 0.806, respectively (**Figure 4**). Under the four future scenarios (SSP126_2050s, SSP585_2050s, SSP126_2090s, SSP585_2090s), the model’s test set AUC values all exceed 0.8. After optimization, the model performance was significantly improved: in both key metrics-the delta AICc value and the omission rate-the optimized model performed better than the default parameter model, demonstrating higher predictive accuracy.

**Figure 5:**
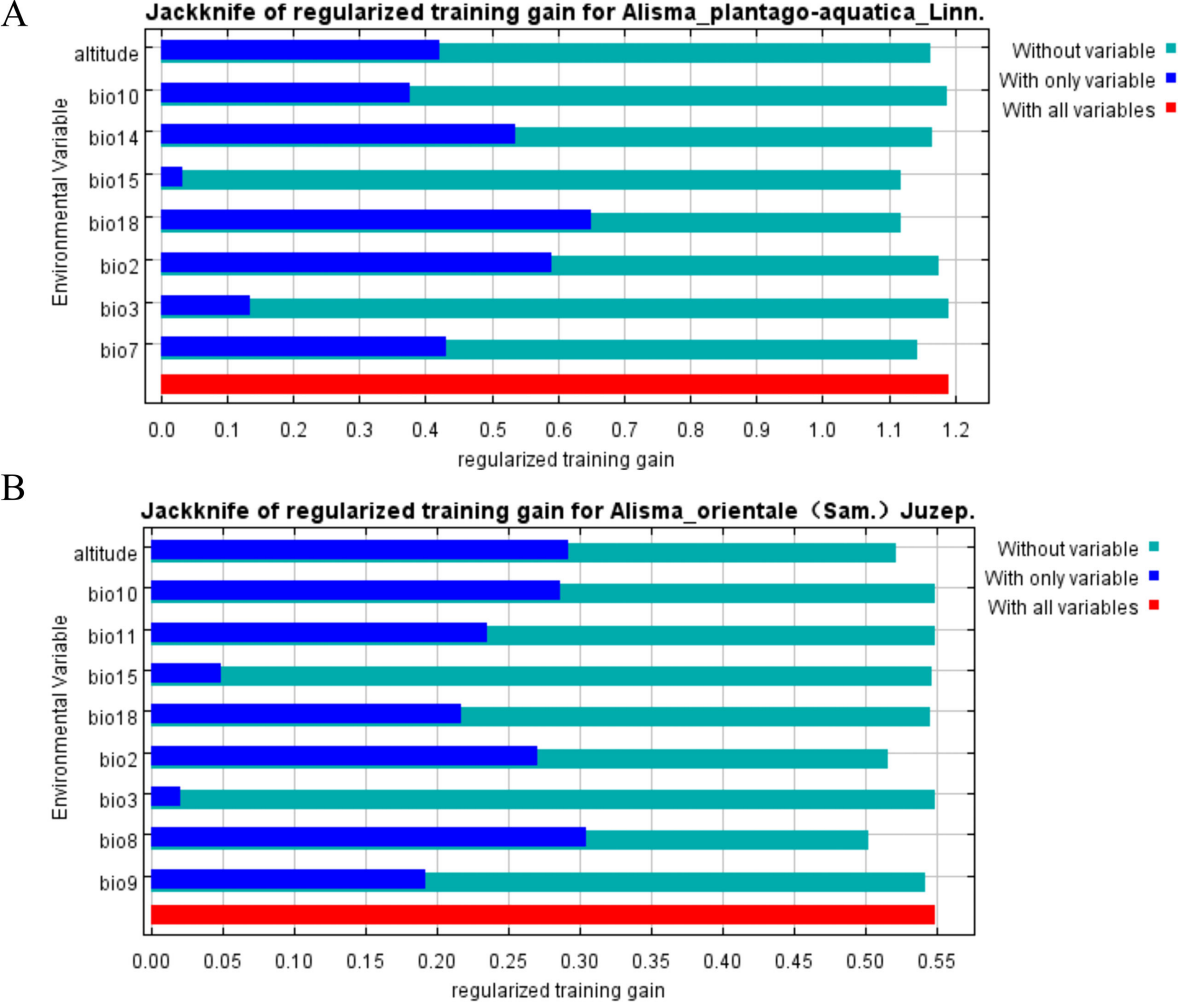
**(A)***A*. *plantago-aquatica* jackknife plot. **(B)***A. orientale* jackknife plot.

According to Jackknife analysis and environmental variable contribution rates (**Figure 5**; **Table 4**), the primary environmental factors contributing over 85% to the potential suitable distribution zones of *A. plantago-aquatica* include: Precipitation of driest month (bio14, 29.3%), Precipitation of warmest quarter (bio18, 20.3%), Altitude (19.4%), Mean diurnal temperature range (bio2, 13.9%), and Precipitation seasonality (bio15, 8.1%). These five factors together account for 91.0% of the total percentage contribution, with a permutation importance of 91.7%. These results indicate that precipitation is a critical environmental factor influencing the suitability of distribution for *A. plantago-aquatica.* Among the five primary influencing factors identified, precipitation-related factors accounted for three, with their cumulative contribution rate reaching as high as 57.7%. Meanwhile, the primary environmental factors contributing over 85% to the potential suitable cultivation zones for *A. orientale* include: Altitude (35.4%), Precipitation of warmest quarter (bio18, 24%), Mean diurnal temperature range (bio2, 14%), Mean temperature of driest quarter (bio9, 10.5%), and Mean temperature of warmest quarter (bio10, 8.7%). The combined percentage contribution of these five factors reached 92.6%, with a permutation importance of 86.9%. This indicates that, apart from altitude, temperature is the key environmental factor regulating the growth of *A. orientale.* Among the five primary factors influencing its growth, three are temperature-related, collectively accounting for 33.2% of the cumulative contribution rate.

**Table 4:** Contribution rates of environmental variables to the model.

Species	Variable	Percent contribution	Permutation importance
*A. plantago-aquatica*	bio14	29.3	6.5
	bio18	20.3	19.7
	altitude	19.4	45.3
	bio2	13.9	10.3
	bio15	8.1	9.9
	bio7	6.2	7.4
	bio10	2	0.4
	bio3	1	0.5
*A. orientale*	altitude	35.4	48.2
	bio18	24	3.2
	bio2	14	12.4
	bio9	10.5	23.1
	bio10	8.7	0
	bio8	4.6	10.8
	bio11	2.5	1.5
	bio15	0.2	0.7
	bio3	0	0.1

**Figure 6:**
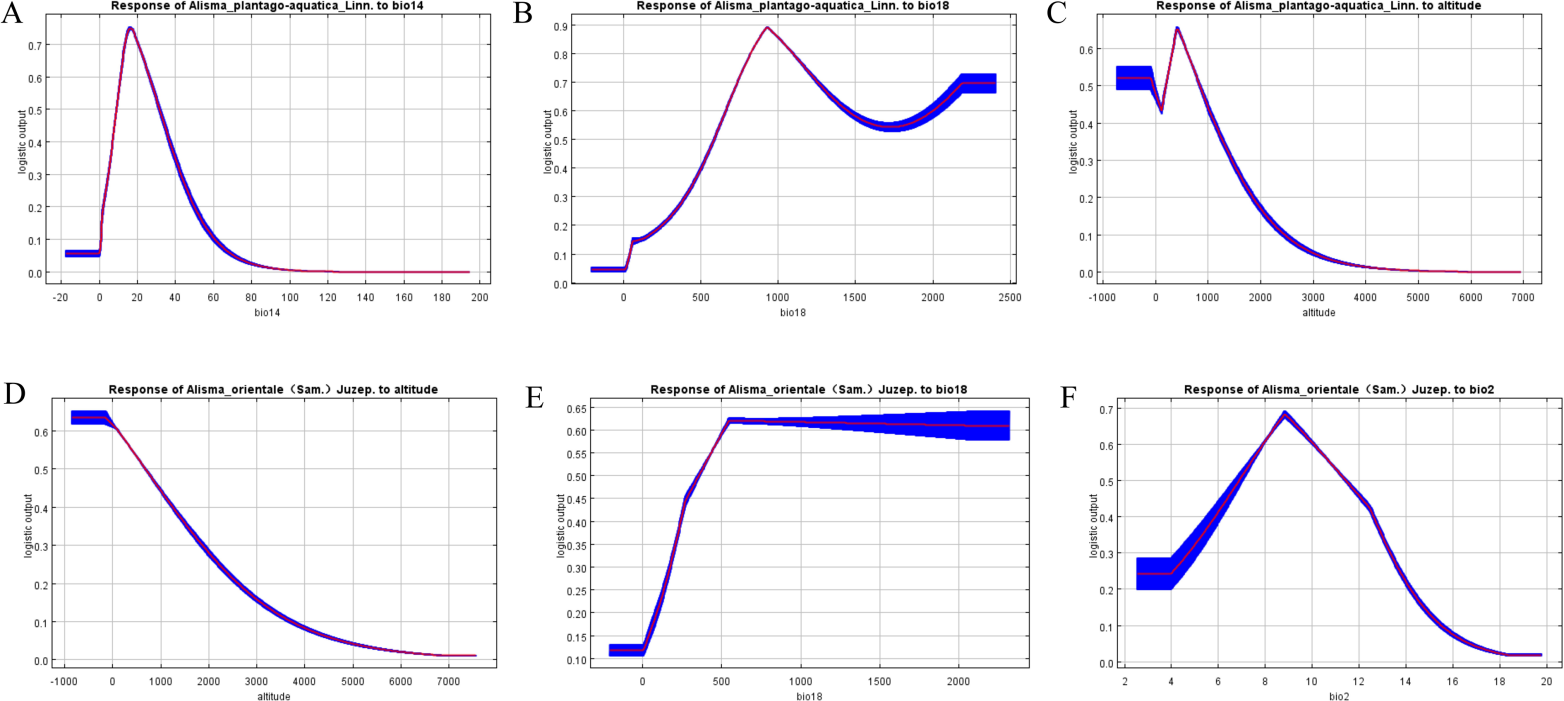
**(A–C)** response curves of major environmental factors for *A. plantago-aquatica.***(D–F)** response curves of major environmental factors for *A. orientale*.

Based on the contribution rate analysis, response curves for the top three environmental variables were plotted (**Figure 6**). Results indicate that the Precipitation of the driest month (bio14) of *A. plantago-aquatica* has the most significant impact on its high-suitability areas, with corresponding precipitation ranging from approximately 11–26 mm. Second is the Precipitation of warmest quarter (bio18). When precipitation ranges between 660–1440 mm or exceeds 2020 mm, it is most suitable for the growth of *A. plantago-aquatica.* Additionally, elevation is a critical factor with high permutation importance; and the optimal elevation range for *A. plantago-aquatica* growth is approximately 330–570 meters. This indicates that *A. plantago-aquatica* prefers humid, low-elevation, and warm eastern habitats. For *A. orientale*, altitude is the key irreplaceable factor influencing its high-suitability areas, which must be below 108 meters. Furthermore, the Precipitation of warmest quarter (bio18) must exceed 517 mm, and the Mean diurnal temperature range (bio2) must fall between 8-10°C. In summary, altitude, Precipitation of warmest quarter (bio18), and Mean diurnal temperature range (bio2) are the three major environmental factors jointly shaping the high-suitability regions for both *A. plantago-aquatica* and *A. orientale*.

### 3.2 Current and future potential distribution area prediction

**Figure 7:**
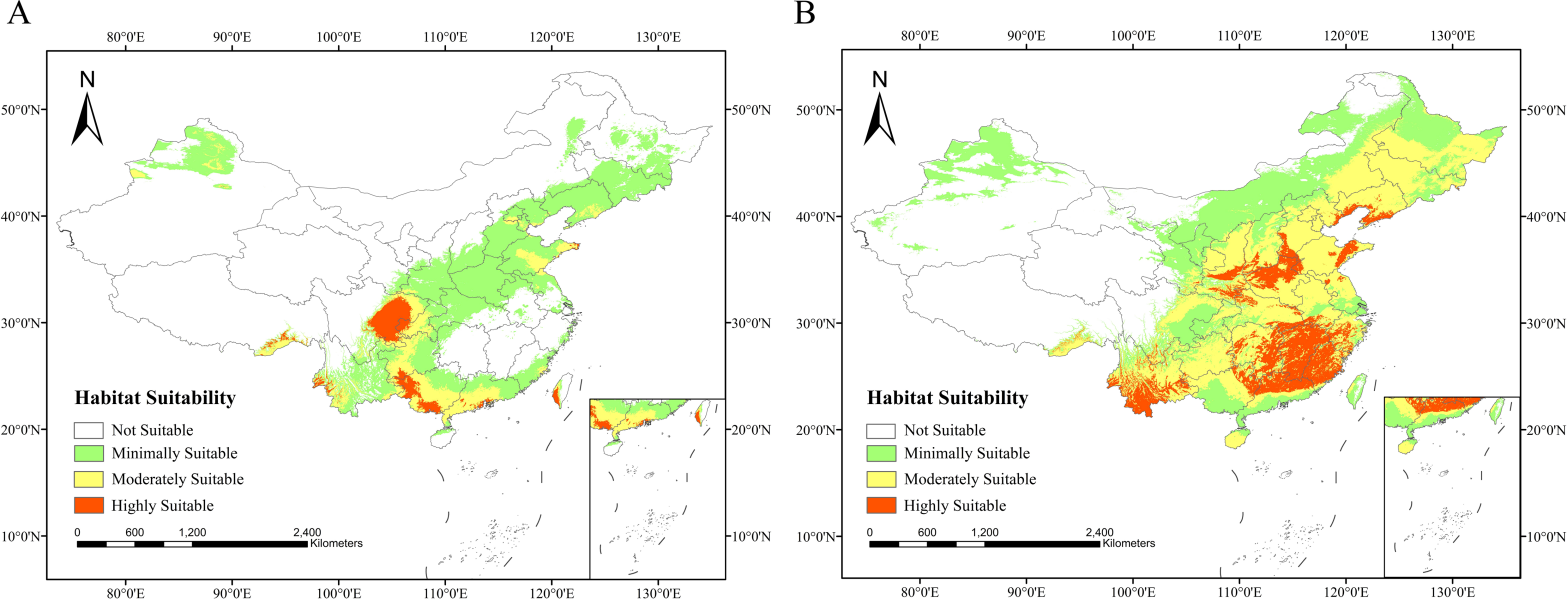
**(A)** Current suitable habitat map for *A*. *plantago-aquatica.***(B)** Current suitable habitat map for *A*. *orientale*.

**Figure 8:**
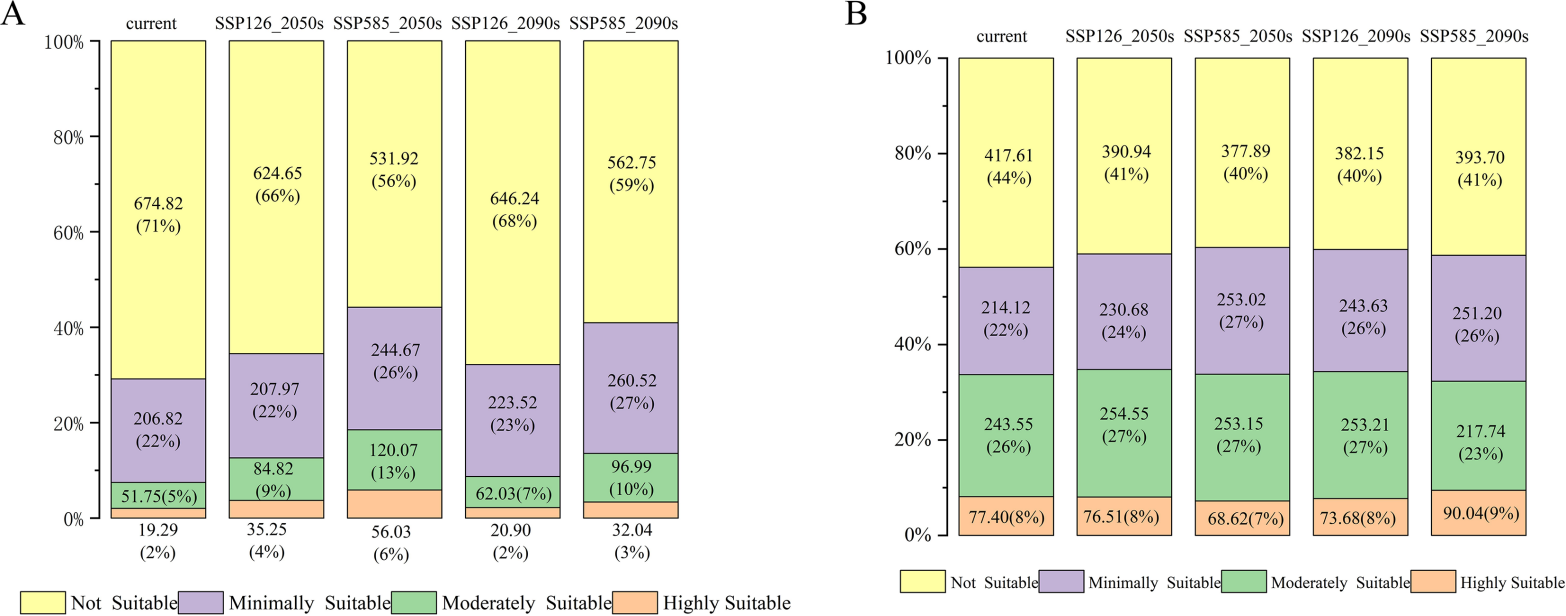
**(A)** Current and future suitable area for *A*. *plantago-aquatica* (×10^4^ km^2^). **(B)** current and future suitable area for *A*. *orientale* (×10^4^ km^2^).

Based on predictions from the optimized MaxEnt model (**Figure 7**), the suitable distribution areas of *A. plantago-aquatica* and *A. orientale* under current climatic conditions were classified into four suitability levels. The suitable habitats of *A. plantago-aquatica* are primarily distributed across eastern Sichuan, western Chongqing, southern and western Guangxi, southern Guangdong, southern Guizhou, and parts of Shandong. Among these, the highly suitable habitat covers an area of 19.29 × 10^4^ km^2^. The suitable habitats distribution of *A. orientale* is relatively extensive, covering 20 provinces including Fujian, Jiangxi, Yunnan, and Henan. The core highly suitable area is primarily concentrated in Fujian, Jiangxi, southern Yunnan, northern Guangdong, northern Henan, and eastern Hunan, with a total area of 90.04 × 10^4^ km^2^ (**Figure 8** and **Table 5**). The total area distributed nationwide is 952.68 × 10^4^ km^2^, which is approximately 0.8% lower than the official land area. This discrepancy stems from simplifications in the base map and operations and calculations within the ArcGIS software, and does not affect the statistical accuracy of the area. Currently, the cultivated area of *A. plantago-aquatica* in its primary production regions spans approximately 0.0053 × 10^4^ km^2^, with *A. orientale* covering about 0.0013 × 10^4^ km^2^. The MaxEnt model predicts significantly larger areas of highly/moderately suitable habitats than the actual cultivation scale, indicating that its predictions hold reference value. Moreover, no obvious bias was observed where “predicted high-suitability areas lacked actual cultivation”. This disparity reflects that the current planting layout has yet to fully utilize ecologically suitable resources, indicating potential for rational expansion. However, the expansion of *A. plantago-aquatica* plant cultivation is constrained not only by ecological conditions but also by multiple factors including market demand, land costs, policy support, labor conditions, and traditional farming practices. Therefore, in subsequent industrial planning, the high-suitability areas identified by the model can serve as a key reference. Priority should be given to regions with strong ecological suitability and low land costs. By adjusting planting scale in response to market dynamics, this approach facilitates the synergistic development of ecological adaptation and economic viability, thereby enhancing industrial competitiveness and sustainable development capacity.

**Table 5:** Current and future suitable area (×10^4^ km^2^).

Species	Different periods	Highly Suitable	Moderately Suitable	Minimally Suitable	Not Suitable
*A. plantago-aquatica*	current	19.29	51.75	206.82	674.82
	SSP126_2050s	35.24	84.82	207.97	624.65
	SSP585_2050s	56.03	120.07	244.67	531.92
	SSP126_2090s	20.90	62.03	223.52	646.24
	SSP585_2090s	32.04	96.99	260.92	562.75
*A. orientale*	current	77.40	243.55	214.12	417.61
	SSP126_2050s	76.51	254.55	230.68	390.94
	SSP585_2050s	68.62	253.15	253.02	377.90
	SSP126_2090s	73.69	253.21	243.63	382.15
	SSP585_2090s	90.04	217.74	251.21	393.70

**Figure 9:**
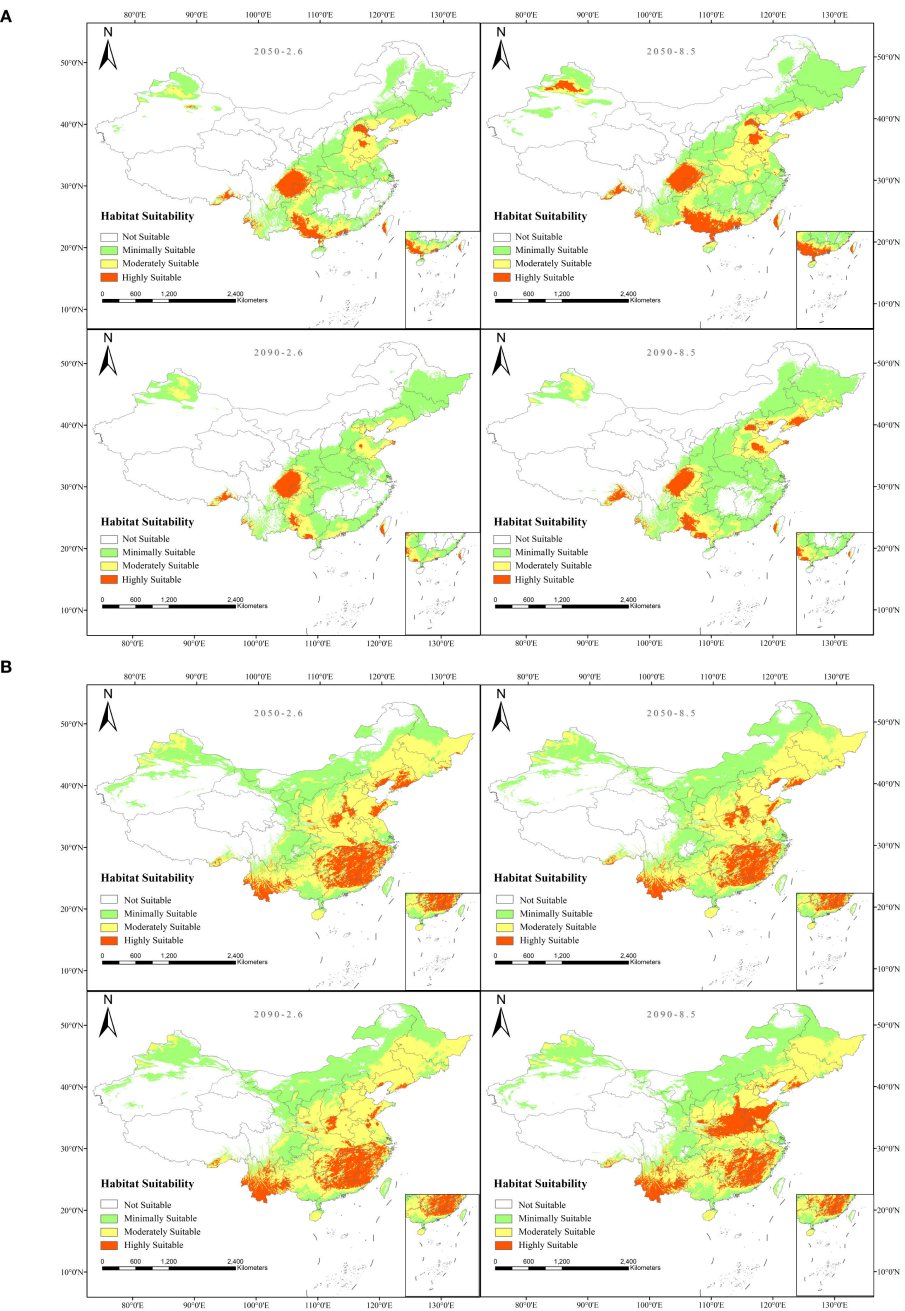
**(A)** Future suitable distribution area map for *A*. *plantago-aquatica*. **(B)** future suitable distribution area map for *A*. *orientale*.

The projected results based on four future climate scenarios (SSP126_2050s, SSP585_2050s, SSP126_2090s, SSP585_2090s) are shown in **Figure 9**. The total area of suitable habitats for *A. plantago-aquatica* has generally shown a trend of increasing first and then decreasing. The primary distribution areas in the future will include eastern Sichuan, western Chongqing, southern and western Guangxi, southern Guangdong, and southern Guizhou, extending to Beijing, Tianjin, southern Liaoning, and Shandong. Notably, under the SSP585 scenario in the 2050s, the area of highly suitable habitat reaches a peak of 56.03 × 10^4^ km^2^. Similarly, the suitable habitat area for *A. orientale* also shows a trend of increasing first and then decreasing. In the future, it will be primarily distributed across Fujian, Jiangsu, Yunnan, Zhejiang, Hunan, northern Guangdong, Hubei, Shandong, and Liaoning provinces. Under the SSP585 scenario in the 2090s, the area of highly suitable habitat reached a maximum of 90.04 × 10^4^ km^2^. Overall, the results suggest that compared to the present, the suitable distribution areas for *A. plantago-aquatica* and *A. orientale* will show an increasing trend under both scenarios (SSP126 and SSP585) during the two future periods (2050s and 2090s). The two selected scenarios, SSP1-2.6 and SSP5-8.5, represent the extreme ends of future climate projections. As AR thrives in warm and humid climates, comparing these two climate scenarios helps to highlight the influence of limiting factors such as temperature and precipitation on its growth.

### 3.3 Trends in the expansion and contraction of suitable distribution areas and centroid shifts for *A. plantago-aquatica* and *A. orientale*

To visually illustrate the spatial dynamics of the suitable distribution areas for *A. plantago-aquatica* and *A. orientale* under climate change, we overlaid their potential distributions under current and future climate scenarios to quantify the trends in suitable area expansion and contraction (**Figure 10**). Analysis results indicate that the spatial variation characteristics of *A. plantago-aquatica* remain relatively consistent across four future climate scenarios. Overall, the suitable area exhibits an increasing trend, with the distribution range shifting northwestward. Newly gained suitable areas are mainly located in eastern Sichuan, western Chongqing, central and southern Guangxi, central Guangdong, as well as parts of Shandong, Beijing, Tianjin, and Liaoning. In contrast, the loss of suitable areas is relatively limited, primarily occurring in southeastern Sichuan, southern and western Guangxi, and parts of Guangdong. Similarly, the suitable area for *A. orientale* also shows a gradual increasing trend. It is particularly noteworthy that under the SSP585 scenario in the 2090s, the increase is most significant, with newly added areas mainly concentrated in southern Henan, central-southern Shandong, and northern Anhui.

**BFigure 10:**
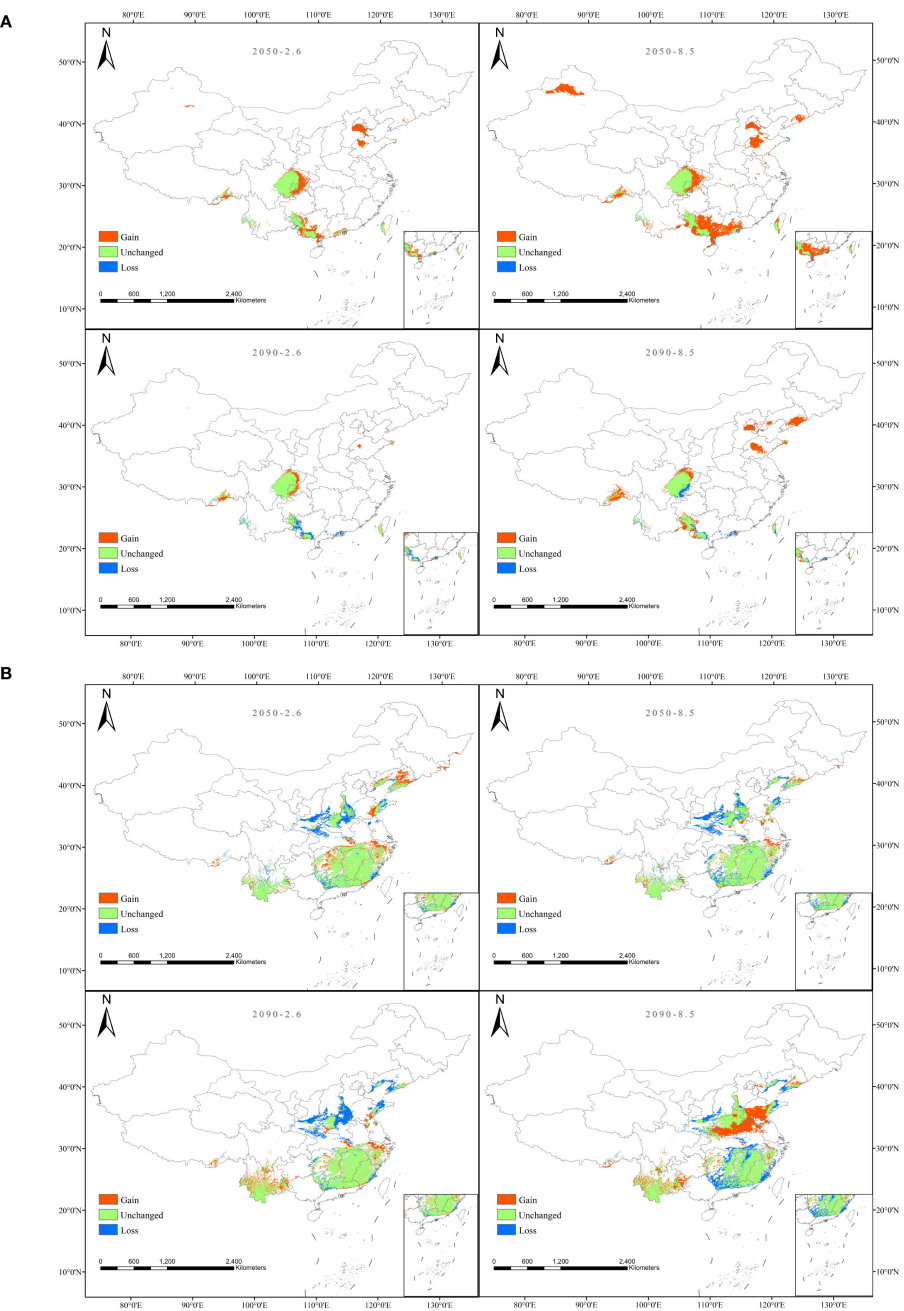
**(A)** Trend chart of changes in the high-suitability area for *A*. *plantago-aquatica*. **(B)** trend chart of changes in the high-suitability area for *A*. *orientale*.

**Figure 11:**
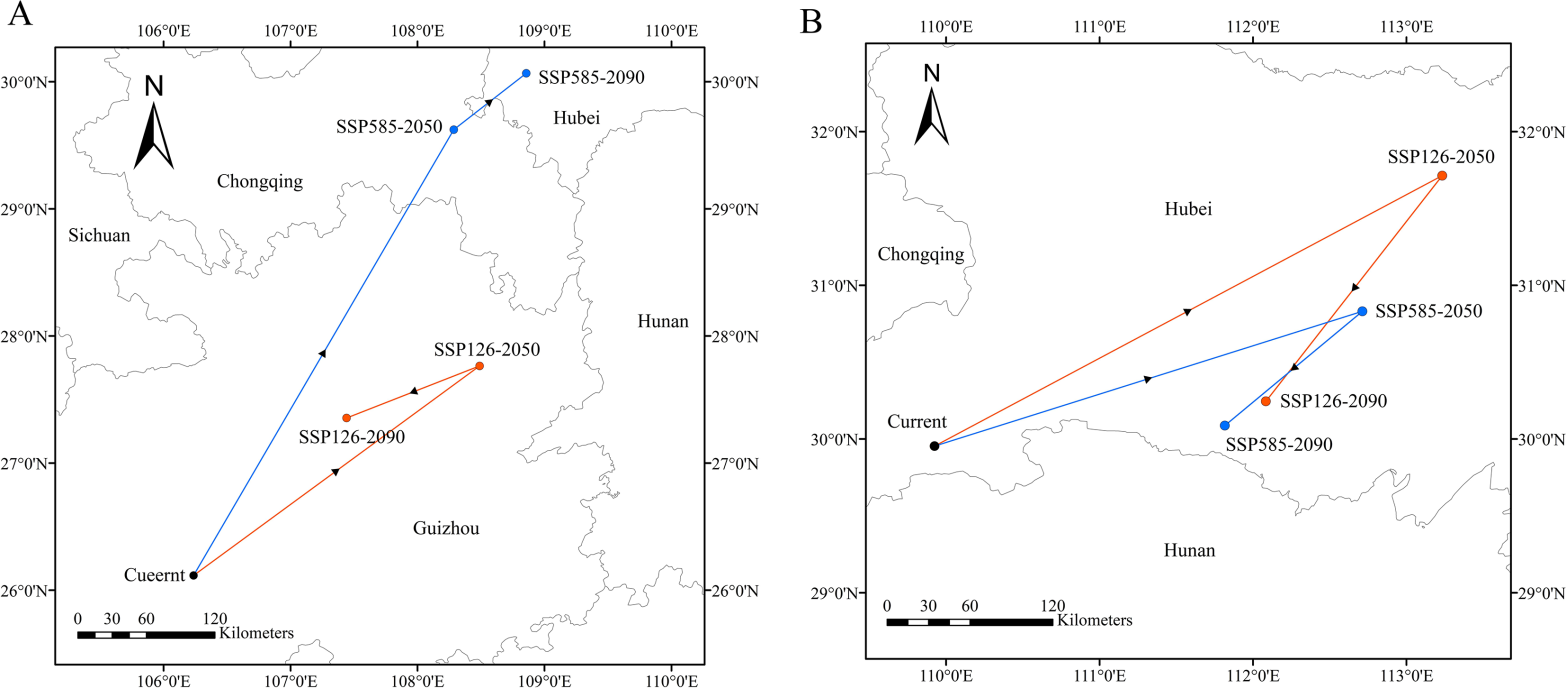
**(A)** Shift of the centroid for the high-suitability area of *A. plantago-aquatica*. **(B)** shift of the centroid for the high-suitability area of *A*. *orientale*.

**Figure 11** shows the trend of the centroid locations of the high-suitability areas for *A. plantago-aquatica* and *A. orientale* under climate change, as mapped using the SDMtoolbox. Under current climatic conditions, the center of the optimal distribution areas for *A. plantago-aquatica* is located at 106.24°E and 26.12°N (Guizhou Province). Under the SSP126 scenario, its center of mass shifted northeastward to 108.49°E, 27.76°N (Guizhou Province) in the 2050s, and southwestward to 107.44°E, 27.35°N (Guizhou Province) in the 2090s. Under the SSP585 scenario, the centroid continues to move northeastward, reaching 108.29°E, 29.62°N (Chongqing Municipality) in the 2050s and 108.86°E, 30.07°N (Hubei Province) by the 2090s. For *A. orientale*, the current distribution centroid is located at 109.93°E, 29.95°N (Hubei Province). Under the SSP126 scenario, its center of mass first shifts northeastward, reaching 113.23°E, 31.72°N in the 2050s, then migrates southwestward, reaching 112.08°E, 30.25°N in the 2090s. Similarly, under the SSP585 scenario, the center of mass also exhibits a migration pattern shifting first northeastward (located at 112.71°E, 30.83°N in the 2050s) and then southwestward (located at 111.82°E, 30.09°N in the 2090s), with the center of mass remaining within Hubei Province throughout both the present and future periods. In summary, under both current and future climate change scenarios, the distribution centers of *A. plantago-aquatica* and *A. orientale* will shift toward the northeast (higher latitudes), albeit along distinct migration paths. Notably, the migration of *A. plantago-aquatica* under the SSP585 scenario is projected to be particularly persistent.

### 3.4 Results of determining size, color, and active ingredient content in AR medicinal materials

The individual weight, length, diameter, and powder color values (L^*^, a^*^, b^*^) of AR from different producing areas are presented in **Table 6** The coefficients of variation (*CV* %) for morphological characteristics of AR from different origins ranged from 2.82% to 81.18%. Among these, the *CV* for individual weight and the a^*^ value of powder color reached 34.65% and 81.18%, respectively, indicating significant variation in size and color among AR specimens from different production areas. Additionally, the results of determining the content of extracts, total triterpenoids, AD, AC, 23-AAC, 24-AAA, AB, 23-AAB, and 11-DAB in AR from different origins are summarized in **Table 7** The coefficient of *CV* for active components in AR from different origins ranged from 19.01% to 49.14%. Specifically, the coefficients of variation for AD, AC, 23-AAC, and 11-DAB exceeded 30%, indicating that, similar to morphological variations, significant differences exist in the accumulation of pharmacologically active components among AR from different origins. To further clarify the significance of the aforementioned differences, analyses were conducted on their morphological characteristics and pharmacodynamic component indicators. Results indicate that among morphological characteristics, Z13 (Gangnan, Guangxi) -origin AR significantly outperformed other regions in single weight (45.37 g), length (61.07 mm), and diameter (46.38 mm), indicating the largest individual size. Meanwhile, Z11 (Jingyang, Sichuan) demonstrated the highest a* value (2.37) and b* value (19.19), revealing pronounced color differences. In terms of pharmacodynamic components, Z8 (Emeishan, Sichuan) showed the highest AC content (0.0383%), Z11 exhibited the highest 11-DAB content (0.0628%), and Z3 (Wutongqiao, Sichuan) recorded the highest 23-AAC content (0.0183%). Additionally, Z3 also demonstrated the highest total triterpenoid content (2.87%) and AB content (0.5306%), indicating a significant overall advantage in the accumulation of pharmacodynamic components. In contrast, most of the effective components in Z12 (Dianjiang, Chongqing) were at relatively low levels, reflecting poor quality performance.

**Table 6:** Quantitative measurement results of six visual characteristics for AR from different origins.

Sample	Weight (g)	Length (mm)	Diameter (mm)	powder color values L^*^	powder color values a^*^	powder color values b^*^
Z1	19.72	53.91	35.17	89.61	0.27	12.66
Z2	19.72	54.23	36.44	90.03	0.03	12.52
Z3	20.94	56.42	36.45	88.1	0.31	13.96
Z4	20.35	50.92	34.50	86.53	0.44	14.72
Z5	22.17	50.72	37.88	86.67	1.24	17.22
Z6	20.79	56.37	34.91	82.67	2.41	18.07
Z7	18.11	44.06	37.38	85.2	1.86	17.63
Z8	21.23	52.65	35.33	85.37	1.44	17.94
Z9	17.51	50.58	35.93	85.19	1.06	16.6
Z10	17.02	46.21	34.25	89.97	0.03	11.95
Z11	16.53	46.72	34.46	82.7	2.37	19.19
Z12	16.98	44.43	34.72	85.42	1.93	17.85
Z13	45.37	61.07	46.38	88.87	0.4	13.06
Z14	30.54	56.88	40.71	85.99	0.8	14.86
*CV*/%	34.5	9.87	8.91	2.82	81.18	15.83

**Table 7:** Content of nine medicinal components in AR from different regions (%).

Sample	extracts	total triterpenoids	AD	AC	23-AAC	24-AAA	AB	23-AAB	11-DAB
Z1	18.92	2.61	0.0020	0.0078	0.0048	0.2170	0.4081	0.2154	0.0483
Z2	17.51	2.67	0.0021	0.0140	0.0076	0.2410	0.4888	0.2375	0.0414
Z3	15.04	2.87	0.0037	0.0319	0.0183	0.2734	0.5306	0.2520	0.0504
Z4	11.17	2.85	0.0046	0.0099	0.0060	0.3103	0.5060	0.2598	0.0618
Z5	10.34	2.96	0.0056	0.0334	0.0183	0.2245	0.4971	0.2496	0.0477
Z6	10.80	2.08	0.0054	0.0275	0.0154	0.1454	0.1952	0.1470	0.0190
Z7	12.89	1.93	0.0046	0.0192	0.0125	0.1499	0.2583	0.1587	0.0239
Z8	11.62	1.92	0.0047	0.0383	0.0223	0.2069	0.3594	0.2195	0.0375
Z9	11.78	2.86	0.0043	0.0209	0.0123	0.2974	0.3811	0.2109	0.0305
Z10	17.73	2.84	0.0028	0.0111	0.0067	0.2573	0.4622	0.2310	0.0533
Z11	16.22	2.77	0.0019	0.0079	0.0060	0.2543	0.5195	0.3516	0.0628
Z12	9.29	1.44	0.0069	0.0199	0.0124	0.1629	0.2353	0.1591	0.0266
Z13	10.59	2.34	0.0029	0.0248	0.0119	0.1860	0.2966	0.1357	0.0245
Z14	14.26	2.27	0.0022	0.0151	0.0100	0.2216	0.3113	0.1525	0.0281
*CV*/%	23.48	19.01	40.75	49.14	45.44	22.88	29.55	27.66	36.72

### 3.5 Soil factor determination results for AR

The physicochemical properties of 14 soil samples from different producing areas (**Supplementary Table S2**) indicate that the soil pH ranges from 5.17 to 7.47 (mean 6.29, *CV* = 10.62%), demonstrating slightly acidic to neutral characteristics overall, which is consistent with previous studies. Specifically, significant differences exist in fertility indicators such as organic matter and total phosphorus content among soils from different origins. Specifically, significant differences exist in fertility indicators such as organic matter and total phosphorus content among soils from different origins. Additionally, the coefficients of *CV* for the 23 measured soil factors ranged from 10.62% to 161.41%. Only soil pH exhibited low variability (*CV* = 10.62%), while the remaining 22 factors showed high coefficients of variation. Notably, urease activity displayed the greatest variability (*CV* = 161.41%), ranging from a minimum of 0.0004 mg/(g·24 h) to a maximum of 0.631 mg/(g·24 h). According to the classification of variation levels, all physicochemical indicators-including OM, TP, AP, available copper, available zinc, available iron, available manganese, available molybdenum, exchangeable calcium, exchangeable magnesium, neutral phosphatase, catalase, polyphenol oxidase, sucrase, and urease-exhibited strong variability (*CV* > 40%), except pH, TN, TK, AN, AK, available boron, acid phosphatase activity, and neutral protease activity. Among these, urease showed the highest coefficient of variation, reaching 161.41%.

### 3.6 Response analysis of environmental factors on the morphological traits and medicinal components of AR

#### 3.6.1 Spearmans’ correlation analysis

**Figure 12:**
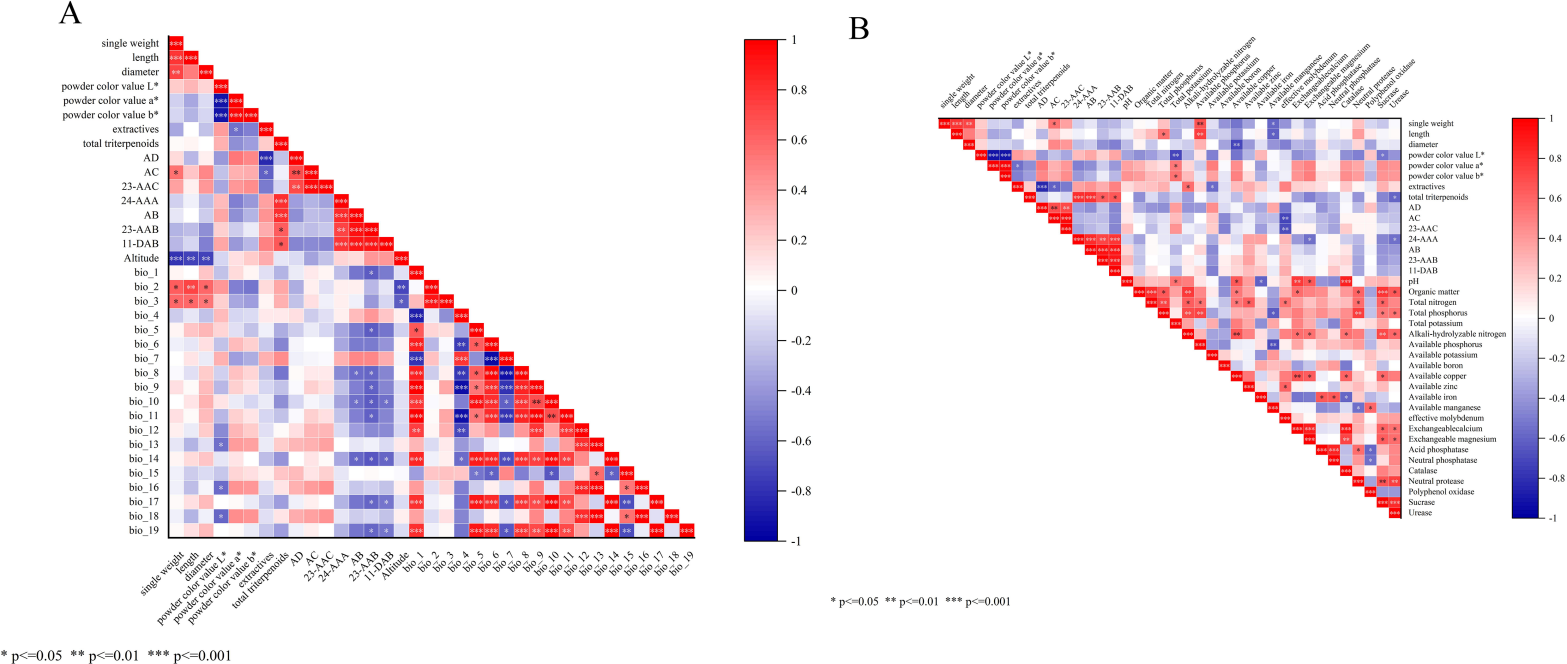
**(A)** Correlation diagram between AR quality and climatic factors. **(B)** correlation diagram between AR quality and soil factors.

Climate change not only affects the expansion or contraction of suitable habitats for medicinal plants, but also significantly influences the formation and accumulation of desirable morphological traits and bioactive components through growing region environmental factors such as climate and soil conditions. Based on the latitude and longitude coordinates of sampling points, this study extracted 19 bioclimatic variables (bio1-bio19) covering temperature and precipitation, combined with elevation data recorded during sampling. Origin 2025 software was employed to conduct Spearman correlation analyses between environmental factors and 15 quality evaluation indicators for AR, including 6 appearance traits and 9 active constituents (**Figure 12**, Data 1 and Data 2).

The results indicate that the quality of AR medicinal material is influenced by multiple climatic factors acting in concert. Regarding its external quality, altitude showed a highly significant negative correlation with individual weight (*P* < 0.001) and a significant negative correlation with length and diameter (*P* < 0.01). Mean diurnal temperature range (bio2) showed a significant positive correlation with length (*P* < 0.01) and a positive correlation with individual weight and diameter (*P* < 0.05). Isothermality (bio3) showed positive correlations with individual weight, length, and diameter (*P* < 0.05). Additionally, Precipitation of wettest month (bio13), Precipitation of wettest quarter (bio16), and Precipitation of warmest quarter (bio18) showed a negative correlation with the L^*^ value of powder color (*P* < 0.05). This indicates that as precipitation increases, powder absorbs moisture, leading to increased light scattering due to caking or agglomeration. Consequently, the L^*^ value decreases, resulting in a darker color. Regarding the active ingredient content, Mean temperature of wettest quarter (bio8), Mean temperature of warmest quarter (bio10), and Precipitation of driest month (bio14) showed a negative correlation with AB content (*P* < 0.05). Annual mean temperature (bio1), Max temperature of warmest month (bio5), Mean temperature of wettest quarter (bio8), Mean temperature of driest quarter (bio9), Mean temperature of warmest quarter (bio10), Mean temperature of coldest quarter (bio11), Precipitation of driest month (bio14), Precipitation of driest quarter (bio17), and Precipitation of coldest quarter (bio19) were negatively correlated with 23-AAB (*P* < 0.05). Mean temperature of warmest quarter (bio10), Precipitation of driest month (bio14), Precipitation of driest quarter (bio17), and Precipitation of coldest quarter (bio19) also showed negative correlations with 11-DAB (*P* < 0.05). In summary, altitude, Mean diurnal temperature range (bio2), and Isothermality (bio3) are key environmental factors influencing the morphological traits of AR. Meanwhile, factors such as Mean temperature of wettest quarter (bio8), Mean temperature of warmest quarter (bio10), and Precipitation of driest month (bio14) play crucial roles in the formation and accumulation of its medicinal components.

By examining the response relationships between 23 soil factors and 15 medicinal herb quality indicators, the results indicate that soil factors significantly influence the quality of AR medicinal herbs. In terms of morphological characteristics, AP showed a significant positive correlation with individual weight and length (*P* < 0.01). Available copper exhibited a significant negative correlation with diameter (*P* < 0.01). TK content showed a significant negative correlation (*P* < 0.01) with the L^*^ value of powder color, and a positive correlation (*P* < 0.05) with the a^*^ and b^*^ values of powder color. TN showed a positive correlation with length (*P* < 0.05). Available manganese exhibited a negative correlation with individual weight and length (*P* < 0.05). Sucrosease activity demonstrated a negative correlation with powder color value L^*^ (*P* < 0.05). Furthermore, regarding the active pharmaceutical ingredient, effective molybdenum exhibited a significant negative correlation with AC and 23-AAC (*P* < 0.01). AN and AK showed positive or negative correlations with leachate (*P* < 0.05). Urease activity exhibited negative correlations with total triterpenoids and 24-AAA (*P* < 0.05). Exchangeable magnesium was negatively correlated with 24-AAA (*P* < 0.05). In summary, TK significantly influenced the color of AR powder (L^*^, a^*^, b^*^); AP affected individual weight and length; available molybdenum affected AC and 23-AAC content; and urease activity affected total triterpenes and 24-AAA content.

#### 3.6.2 Stepwise linear regression analysis

To further identify key environmental factors that significantly influence the accumulation of both the morphological characteristics and bioactive constituents of AR, we established a stepwise linear regression model linking environmental factors to the quality of AR (**Table 8**). The significance level for inclusion in the regression model was set at *P* ≤ 0.05, while the threshold for exclusion was *P* ≥ 0.10. Multicollinearity diagnostics were performed (VIF < 10). The results indicate that altitude exerts a negative effect on the individual weight and diameter of AR, but a positive effect on the content of 23-AAB. Mean diurnal temperature range (bio2) showed a negative correlation with the powder color values a^*^ and b^*^, and a positive correlation with length. Precipitation of driest month (bio14) hurt 11-DAB content. Regarding soil factors, OM showed a positive correlation with individual weight, diameter, and powder color value a^*^. TK exerted a negative influence on individual weight and powder color value L^*^, while exerting a positive influence on powder color value a^*^. The effective copper content negatively affects both particle weight and diameter, while positively influencing the b^*^ value of powder color. Urease positively affects particle diameter and the a^*^ value of powder color, while negatively affecting the L^*^ value of powder color. Available manganese exhibits a negative correlation with length but a positive correlation with AD content. Available molybdenum exerts a negative influence on both AC and 23-AAC content. TN shows a negative correlation with powder color value a^*^. AK correlates negatively with AB content. AN correlates negatively with individual weight, while acid phosphatase also correlates negatively with diameter. In summary, based on the number of medicinal herb quality indicators dominated by environmental factors, the ranking from highest to lowest is as follows: Mean diurnal temperature range (bio2), altitude, OM, TK, available copper, urease activity > available manganese, available molybdenum > Precipitation of driest month (bio14), TN, AK, AN, and acid phosphatase.

**Table 8:** Stepwise regression models for AR quality and climate factors, soil factors.

Relationship	Indicator	Stepwise regression equation	*r* ^2^	*F*	*P*
Quality of AR and climatic factors	*Y_1_*	*Y_1_* = - 7.91*10^-7^ - 0.893*X_1_	0.798	47.272	0.000
	*Y_2_*	*Y_2_* = - 5.142*10^-7^ + 0.72*X_2_	0.518	12.904	0.004
	*Y_3_*	*Y_3_* = - 8.61*10^-8^ - 0.879*X_1_	0.773	40.975	0.000
	*Y_4_*	*Y_4_* = - 2.995*10^-7^ - 0.581*X_2_	0.337	6.107	0.029
	*Y_5_*	*Y_5_* = - 2.72*10^-7^ - 0.619*X_2_	0.383	7.457	0.018
	*Y_6_*	*Y_6_* = 9.692*10^-7^+ 0.643*X_1_	0.414	8.466	0.013
	*Y_7_*	*Y_7_* = -1.43*10^-6^- 0.56*X_3_	0.314	5.484	0.037
Quality of AR and soil factors	*Y_1_*	*Y_1_* = - 6.308*10^-8^ - 0.486*X_4_ + 1.558*X_5_ - 1.205*X_6_ - 0.406*X_7_	0.914	24.042	0.000
	*Y_2_*	*Y_2_* = 4.068*10^-7^ - 0.57*X_8_	0.324	5.763	0.033
	*Y_3_*	*Y_3_* = 9.091*10^-7^ - 1.873*X_4_ + 0.857*X_5_ + 1.137*X_9_- 0.577*X_10_	0.915	24.108	0.000
	*Y_4_*	*Y_4_* = 9.479*10^-7^ + 0.939*X_9_ + 0.464*X_7_ - 1.059*X_11_ + 0.694*X_5_	0.852	12.905	0.010
	*Y_5_*	*Y_5_* = -7.14*10^-7^ + 0.539*X_4_	0.291	4.914	0.047
	*Y_8_*	*Y_8_* = 2.496*10^-7^ - 0.651*X_9_ - 0.509*X_7_	0.640	9.783	0.004
	*Y_9_*	*Y_9_* = - 1.86*10^-6^ + 0.61*X_8_	0.372	7.119	0.020
	*Y_10_*	*Y_10_* = 6.684*10^-7^ - 0.645*X_12_	0.416	8.560	0.013
	*Y_11_*	*Y_11_* = 6.605*10^-7^ - 0.642*X_12_	0.412	8.395	0.013
	*Y_12_*	*Y_12_* = - 4.208*10^-7^ - 0.589*X_13_	0.347	6.377	0.027

*Y_1_*, single weight. *Y_2_*, length; *Y_3_*, diameter. *Y_4_*, powder color value a^*^. *Y_5_*, powder color value b^*^. *Y_6_*, 23-AAB. *Y_7_*, 11-DAB. *Y_8_*, powder color value L^*^. *Y_9_*, AD. *Y_10_*, AC. *Y_11_*, 23-AAC. *Y_12_*, AB. *X_1_*, altitude. *X_2_*, bio2. *X_3_*, bio4. *X_4_*, available copper. *X_5_*, OM; *X_6_*, AN. *X_7_*, TK. *X_8_*, available manganese. *X_9_*, urease. *X_10_*, acid phosphatase. *X_11_*, TN. *X_12_*, available molybdenum. *X_13_*, AK.

Additionally, both the individual weight and diameter of AR showed extremely significant correlations with elevation (*r*^2^ values of 0.798 and 0.773, respectively, *P* < 0.000), with elevation explaining approximately 80% of the variation in both traits. The regression *r*^2^ values between the remaining quality indicators and climatic factors ranged from 0.314 to 0.518, all reaching significant levels (*P* < 0.05). The soil factors exhibit a multi-factor synergistic influence pattern. For example, the variations in the available copper and organic matter, among four factors, collectively explain the observed trends (*r*^2^ = 0.914, *P* < 0.000). Similarly, the diameter shows significant correlations with four soil factors (*r*^2^ = 0.915, *P* < 0.000). The models for other quality indicators and soil factors yielded *r*^2^ values ranging from 0.291 to 0.852, all of which were statistically significant (*P* < 0.05). Overall, climatic factors primarily exhibited single-factor correlations, whereas soil factors predominantly involved multi-factor combinations, with the latter providing higher explanatory power for certain quality indicators.

Both Spearman correlation analysis and stepwise linear regression results consistently demonstrate that, among climatic factors, elevation is negatively correlated with the weight and diameter of individual AR. Mean diurnal temperature range (bio2) is positively correlated with tree length, while the Precipitation of driest month (bio14) is negatively correlated with 11-DAB content. Among soil factors, TK showed a negative correlation with the L^*^ value of powder color, while exhibiting a positive correlation with the a^*^ value. Available copper correlated negatively with diameter. Available manganese correlated negatively with length. Available molybdenum correlated negatively with AC and 23-AAC content.

## 4 Discussion

### 4.1 The influence of environmental factors on the optimal distribution areas of *A. plantago-aquatica* and *A. orientale*

From a plant ecology perspective, environmental conditions directly influence plant physiology, distribution, and phenology. Among these, precipitation and temperature are the primary factors influencing most plant growth (Jiang et al., 2020), with temperature being a key determinant of plant growth and development, distribution, and seasonal behavior (Ding et al., 2020). Similarly, within different altitudinal ranges, the intraspecific performance of plants varies with changes in temperature and precipitation (Midolo and Wellstein, 2020). Modern research indicates that temperature and precipitation significantly influence the growth of aquatic plants (Zhang P. et al., 2019), determining the suitable distribution ranges of *Phragmites australis* and *Typha orientalis* (Choi and Lee, 2022). The MaxEnt prediction results of this study indicate that the suitable cultivation areas for both *A. plantago-aquatica* and *A. orientale* are primarily influenced by precipitation, temperature, and altitude. This finding aligns with their biological characteristics as aquatic plants, suggesting that both species thrive in warm, humid subtropical monsoon climates with abundant rainfall (Zhou et al., 2011). At the same time, since elevation is highly correlated with meteorological factors such as temperature and precipitation (Cao et al., 2020), it significantly influences the growth of *A. plantago-aquatica* and *A. orientale*. The predicted results for the optimal distribution zones in this study are consistent with the actual cultivation areas of *A. plantago-aquatica* and *A. orientale* in China, both of which are located in provinces such as Sichuan and Fujian.

However, as the two botanical origins of the medicinal substance derived from AR, the key environmental factors influencing the distribution of *A. plantago-aquatica* and *A. orientale* differ significantly beyond altitude: precipitation for *A. plantago-aquatica* and temperature for *A. orientale.* This discrepancy can be attributed to differences in the dominant climatic constraints imposed by their respective geographical environments. The Sichuan Basin, the core cultivation area for *A. plantago-aquatica*, features a unique basin topography that results in relatively warm winters (weak low-temperature limitation) and tempered summer heat due to frequent cloud cover and fog (relatively weak high-temperature limitation). Consequently, precipitation emerges as the most critical variable influencing water supply for *A. plantago-aquatica*. In contrast, the core growing region of *A. orientale* in northern Fujian features open mountainous or hilly terrain. The region is susceptible to cold air masses in winter and experiences intense heat in summer. However, due to exceptionally abundant rainfall and relatively accessible water management infrastructure, the importance of water-related factors is secondary to temperature in limiting the growth and distribution of *A. orientale*.

On the other hand, environmental factors drive genetic differentiation through natural selection mechanisms, leading to the gradual development of adaptive differences between *A. plantago-aquatica* and *A. orientale* in response to distinct environmental factors over the course of long-term evolution. Specifically, as the dominant species within the genus *Alisma*, *A. plantago-aquatica* typically exhibits high biomass, rapid growth rates, and strong resource competitiveness, which may be related to its sensitivity to precipitation levels. The relatively low genetic diversity in both species indicates (Lan et al., 2024) that the divergence time between *A. plantago-aquatica* and *A. orientale* occurred relatively recently. Furthermore, based on geographic distribution, Zhang and Chen (1994) proposed that *A. orientale* represents a divergent lineage of *A. plantago-aquatica* in East Asia, while Ito and Tanaka (2023) proposed that it originated through sympatric divergence with *A. plantago-aquatica.* This may be attributed to the response of *A. orientale* to temperature factors, leading to the development of distinct ecological strategies. This differentiation further resulted in the emergence of separate strains within the *A. plantago-aquatica*, which subsequently dispersed to form the current distribution range. Additionally, it is worth noting that this differentiation in environmental adaptability may also be significantly influenced by human domestication selection. Herbal textual research indicates that the cultivation history of *A. plantago-aquatica* in the Sichuan Basin dates back to the Ming Dynasty, while large-scale cultivation of *A. orientale* in Fujian began during the Qing Dynasty (Chen et al., 2011; Liu, 2019; Lu et al., 2016). Long-term artificial selection during medicinal cultivation in different regions may have further exacerbated the differences between these two species in terms of environmental factors within suitable cultivation zones.

### 4.2 Changes in spatial patterns and centroid of *A. plantago-aquatica* and *A. orientale*

*A. plantago-aquatica* and *A. orientale* are both perennial aquatic or marsh herbs that thrive in warm, humid subtropical monsoon climates. Their suitable habitats are closely associated with river systems. The findings of this study indicate that under current climatic conditions, *A. plantago-aquatica* is primarily distributed in the eastern and southeastern parts of Southwest China and in South China. In contrast, *A. orientale* has a broader distribution range, encompassing East China, Central China, North China, Northeast China, South China, and the southern regions of Southwest China. These regions encompass the basins of the Yangtze, Pearl, Huai, and Hai Rivers, where a warm and humid climate combined with abundant water flow creates ideal habitats. This is highly consistent with the analysis results based on TCMGIS by Lin et al. (2011). Based on TCMGIS, which indicated that *A. orientale* has a broad suitable distribution zone across 12 provinces, including Sichuan, Guangdong, Guangxi, and Fujian. This also aligns with the actual distribution and cultivation areas of the two plants today. However, due to the relatively high labor and land costs associated with cultivating *A. orientale* in suitable areas, the actual cultivation area has significantly decreased in recent years.

Given that plant geographical distribution is significantly driven by climate, global warming is altering species distribution patterns and suitable habitats (Tewari et al., 2017; Gong et al., 2020). This intensifies plant responses to habitat shifts, with some species’ habitats expanding in warmer climates (Auffret and Svenning, 2022). This study indicates that the suitable cultivation areas for *A. plantago-aquatica* and *A. orientale* will generally expand in the future. This finding aligns with previous conclusions regarding the impact of climate change on the distribution of medicinal plants, such as *Forsythia suspensa* (Thunb.) Vahl (Wang et al., 2024), *Houttuynia cordata* Thunb (Liu et al., 2021), and *Pulsatilla chinensis* (Bunge) (Wu et al., 2025b). However, some studies also indicate that the future suitable habitats of certain medicinal plants may show a shrinking trend (Yang et al., 2023). Such prediction discrepancies are common in species distribution modeling studies, primarily due to model methodology and parameterization, species-specific ecological traits, and the selection of climate scenarios (Cobos et al., 2019; Elith et al., 2010; Eyring et al., 2016; Pearson et al., 2014; Wiens et al., 2009). Future research aims to reconcile these discrepancies by employing integrated modeling approaches. This involves averaging the results from multiple algorithms and parameter settings to generate more reliable predictive outcomes. Furthermore, experimental research on plant heat tolerance and adaptive potential is urgently needed to further validate the accuracy of model predictions.

The distribution centroids of the highly suitable areas for *A. plantago-aquatica* and *A. orientale* are projected to shift northeastward (i.e., toward higher latitudes) in the future. This finding aligns with the northward expansion predicted by most studies, such as those on *Aconitum leucostomum* (Xu et al., 2024), Nymphaea (Parveen et al., 2022), East Asian wild radish (Han et al., 2023), and *Hippuris vulgaris* (Xu et al., 2025). Taken together, these findings collectively confirm the high sensitivity of medicinal plant distribution patterns to climate change. Therefore, to ensure a continuous supply for clinical medicinal needs, future conservation and cultivation strategies must specifically address the migration trends of medicinal plant species. Moreover, as key semi-aquatic plants in wetland ecosystems, *A. plantago-aquatica* and *A. orientale* play vital roles in water purification, landscape beautification, and maintaining ecological balance. Changes in their distribution must also be incorporated into ecological conservation considerations.

### 4.3 The influence of environmental factors on the quality of AR

From the perspective of crop cultivation science, even within suitable distribution zones for medicinal plants, specific local environmental factors, such as microclimate, soil properties, and biological communities, act as a “precision reaction vessel” that directly regulates plant growth and development, as well as the synthesis and accumulation of secondary metabolites. These factors ultimately determine both the morphological traits and the intrinsic quality of medicinal materials. Therefore, future development strategies for traditional Chinese medicinal herb agriculture must closely integrate macro-level ecological adaptability predictions (answering “where the plant can grow”) with micro-level physiological cultivation regulation (answering “where it grows best”). Accordingly, we conducted a comprehensive study and analysis of the correlation between the 43 environmental factor indicators, including climate and soil conditions of the main production areas, and the 15 quantitative quality indicators of the current mainstream variety of *A. plantago-aquatica* medicinal material. This research was conducted from two dimensions: the external characteristics of the medicinal material and its primary active constituents. The objective is to identify the key environmental factors essential for producing high-quality AR medicinal material, thereby laying a solid foundation for subsequent research on quality zoning of AR medicinal material.

Through correlation and stepwise regression analyses, this study revealed that environmental factors such as altitude, temperature, and precipitation significantly influence the quality of AR. This result aligns closely with the MaxEnt model simulations, confirming the reliability of the predictions presented in this study. Recent research has shown that temperature participates in the biosynthetic and regulatory processes involved in the formation of AR quality. Specifically, temperature can influence the accumulation of secondary metabolites by regulating the activity of key rate-limiting enzymes in the triterpenoid biosynthesis pathway (Lu et al., 2020). Effective accumulated temperature showed a significant positive correlation with the content of 23-AAB and 23-AAC in both *A. plantago-aquatica* and *A. orientale* (Lang et al., 2023). The research teams’ preliminary investigation also revealed that high temperatures can cause tuberous growths on *A. plantago-aquatica*, indicating that temperature does indeed affect the morphological characteristics of AR medicinal materials. Additionally, Zhu et al. (2019) indicated that precipitation during the early growing season (March-June) significantly influences the accumulation of essential oils in various medicinal plants, including *Atractylodes lancea*, *Angelica sinensis*, *Curcuma longa*, and *Atractylodes macrocephala*. Polyphenolic compounds in *Rheum tanguticum* Maxim. ex Balf. showed a negative correlation with annual precipitation and rainfall during the wettest season (Yang et al., 2021). Regarding altitude factors, the content of active compounds in Ziziphi Spinosae Semen increases with rising altitude within the range of 60–500 meters (Liu Q. Y et al., 2025). Ephedrine and pseudoephedrine also exhibit altitude-related variations in two *Ephedra* species (*Ephedra gerardiana* and *Ephedra saxatilis*) (Lu et al., 2023). Golian et al. (2025) found that at low altitudes, *Lavandula x intermedia* exhibited the highest essential oil content, with linalool reaching up to 27.98%.

This study measured 23 soil factors across 14 production areas in China, revealing substantial variation coefficients among these factors. This indicates significant regional differences in soil conditions, providing a foundation for explaining the spatial variation in the quality of AR medicinal materials. Based on the results of correlation analysis and stepwise regression, key soil factors can be identified for evaluating soil fertility and influencing the quality of AR. Existing literature indicates that soil OM, TN, and AK show positive correlations with the active components of AR (such as 11-DAB, AB, and 23-AAB) (Yang, 2025). This finding supports the conclusions of the present study, further confirming that the quality of AR herbal materials is influenced by the synergistic effects of multiple factors. Soil factors do not act independently but jointly regulate the synthesis and accumulation of active components in medicinal materials through synergistic or antagonistic mechanisms, indicating that soil exerts a systemic influence on the formation of medicinal material quality (Su et al., 2023; Yang et al., 2024b). This study expands the dimensions of soil evaluation by introducing indicators of available elements and soil enzyme activity, building upon existing literature that typically focuses on soil physicochemical properties, inorganic elements, and heavy metal indicators. This approach enhances the systematic nature and reference value of the results. Future research should involve multi-regional sampling and controlled experiments to thoroughly analyze the interactive effects between soil factors and human management practices, and to clarify the specific mechanisms and pathways through which soil factors influence the growth and quality formation of medicinal herbs.

Considering the constraints of the existing soil database in terms of indicator diversity and data comprehensiveness, it is not yet feasible to adequately capture the strong local heterogeneity of soil factors. In addition, the database does not cover data on key indicators closely correlated with medicinal material quality, such as soil enzyme activity. For this reason, soil factors were not included in the analysis for predicting the suitable distribution areas of AR. For example, suitability studies of medicinal plants American ginseng, *Lanxangia tsao-ko*, and four species from North Africa (Zhang X. et al., 2023; He et al., 2024; Mechergui et al., 2025). But in our follow-up research, we precisely collected corresponding soil samples from the major producing regions of AR, and further systematically investigated and clarified the correlation between 23 soil indicators and the quality of AR. This practice had fulfilled the overall research goal of extending from “suitable growing zones” to “high-quality cultivation areas”, and also enabled a more accurate demonstration of how soil properties influence the quality of AR.

In addition, the “bidirectional multidimensional indicator system” proposed in this study can be applied to the cultivation zoning of AR, the diagnosis of quality degradation in traditional production areas, and the early warning of cultivation zone migration under climate change. The practical workflow consists of three steps: first, core indicators are identified through literature review, relevance analysis, and MaxEnt contribution rate screening; second, the model is used to delineate high/medium/low suitability zones; finally, the reliability of results is validated through field cultivation sites.

However, this study has certain limitations. Species distribution models themselves rely on environmental variables and species distribution point data, but currently available data are relatively limited in terms of temporal series, making it difficult to characterize continuous temporal distribution patterns. Despite optimizing model parameters using the Kuenm software package, future global climate change and human interference continue to introduce significant uncertainty into distribution projections. Furthermore, the model does not account for the influence of biological factors such as disease transmission, pest infestations, and symbiotic microbial interactions. These biological elements may significantly affect a species’ actual suitable range and population persistence, thereby limiting the comprehensiveness of the prediction results to some extent. Looking ahead, in addition to increasing sample sizes and continuously improving the accuracy of quality assessments, efforts should also focus on refining species suitability area prediction models by incorporating key factors such as biological interactions.

## 5 Conclusion

As a commonly used traditional Chinese medicine in clinical practice, AR possesses significant economic value and broad market prospects. It is widely cultivated in China with high yields, yet ecological research on AR remains limited, and in some cases, yields contradictory findings. This highlights an urgent need for systematic studies. As one of the essentials of Chinese civilization, the quality of Chinese medicinal materials is defined by both their intrinsic chemical constituents and external morphological characteristics. And “quality evaluation through morphological identification” is a summary of the traditional experience of Chinese medicinal materials, that is, to determine the authenticity and quality of Chinese medicinal materials through the external characteristics such as “form, color, qi, taste, and quality” (Xie, 1994). To address the limitations of prior studies that relied on only one or a few triterpenoids for quality assessment, this research systematically expanded multidimensional chemical indicators and enhanced comprehensive chemical profiling. For the first time, we integrated the traditional approach of appearance-based quality evaluation into a modern scientific framework, translating it into quantifiable and objective metrics. This allows a holistic and multidimensional evaluation of AR quality. In an innovative approach, this study combined the MaxEnt model (optimized using the Kuenm package) with HPLC, while integrating the classical TCM concept of “quality evaluation through morphological identification” with principles of plant ecology and crop cultivation. This integrated approach enabled a systematic analysis of the spatiotemporal distribution patterns of both *A. plantago-aquatica* and *A. orientale* across their current and future suitable habitats in China. From a plant ecology perspective, our work provides the first comprehensive and timely insights into the macro-scale distribution of this medicinal material. Importantly, our study pinpoints specific climatic and soil conditions that determine AR quality at a microscopic level from a crop cultivation standpoint. For practical application, these results will be used as a critical reference for guiding the selection of cultivation zones and facilitating the regionalization of production for AR. The integration of traditional quality assessment principles with modern analytical techniques and ecological modeling establishes a novel paradigm for investigating the effects of ecological factors on the quality of Chinese medicinal materials. This interdisciplinary approach not only provides scientific support for the ecological cultivation and resource conservation of AR, but can also be extended to research on other medicinal plants. It holds profound significance for promoting the sustainable development of the traditional Chinese medicine industry and advancing coordinated ecological and environmental governance. From a practical perspective, it helps optimize agricultural land use, enhance the standardization of medicinal herb quality, and inject new momentum into policy formulation and regional economic development.

## Data Availability

The original contributions presented in the study are included in the article/**Supplementary Material**. Further inquiries can be directed to the corresponding author.
