# Supplementary material for: Prediction of suitable distribution areas for Alismatis Rhizoma based on the MaxEnt model and study on quality-environment correlation
Source: Front Plant Sci. 2026 Jan 30;17:1723386. doi: 10.3389/fpls.2026.1723386 (PMC12901336; doi:10.3389/fpls.2026.1723386)
Supplement: Supplementary file 3 [file Table1.docx]

Supplementary Material

# Supplementary Tables

**Table S1** Variable Contribution Values.

| Species | Variable | Percent contribution | Permutation importance |
| --- | --- | --- | --- |
| *A. plantago-aquatica* | bio14 | **21.7** | **0.4** |
|  | altitude | **15.3** | **26.2** |
|  | bio18 | **11.7** | **12.5** |
|  | bio2 | **8.5** | **13.4** |
|  | bio7 | **6.4** | **1.4** |
|  | bio15 | **5.4** | **6.3** |
|  | bio10 | **2.1** | **2.1** |
|  | bio3 | **0.7** | **0.2** |
| *A. orientale* | altitude | **25.5** | **19.5** |
|  | bio11 | **22.7** | **2.2** |
|  | bio2 | **9.2** | **6.9** |
|  | bio10 | **8.1** | **6.5** |
|  | bio8 | **7.3** | **6.6** |
|  | bio9 | **4.4** | **3.1** |
|  | bio15 | **4.4** | **2.4** |
|  | bio18 | **1.2** | **1.9** |
|  | bio3 | **0.6** | **0.6** |
