# Supplementary material for: Prediction of suitable distribution areas for Alismatis Rhizoma based on the MaxEnt model and study on quality-environment correlation
Source: Front Plant Sci. 2026 Jan 30;17:1723386. doi: 10.3389/fpls.2026.1723386 (PMC12901336; doi:10.3389/fpls.2026.1723386)
Supplement: Supplementary file 4 [file Table2.docx]

Supplementary Material

# Supplementary Tables

Table S2 soil factor content in AR from different origins.

| *CV* (%) | Z14 | Z13 | Z12 | Z11 | Z10 | Z9 | Z8 | Z7 | Z6 | Z5 | Z4 | Z3 | S2 | Z1 | Sample No. |
| --- | --- | --- | --- | --- | --- | --- | --- | --- | --- | --- | --- | --- | --- | --- | --- |
| 110.62 | 5.45 | 5.17 | 6.02 | 6.76 | 5.85 | 5.98 | 6.28 | 6.34 | 7.47 | 6.20 | 6.15 | 7.42 | 6.02 | 6.99 | pH |
| 40.00 | 33.05 | 40.06 | 20.47 | 49.39 | 20.09 | 34.67 | 66.11 | 41.02 | 37.35 | 14.63 | 24.12 | 27.70 | 26.73 | 46.63 | OM (g/kg) |
| 28.71 | 1.61 | 1.68 | 1.04 | 2.47 | 1.40 | 1.86 | 2.39 | 1.40 | 1.84 | 0.82 | 1.63 | 1.40 | 1.60 | 2.34 | TN (g/kg) |
| 51.18 | 1.03 | 0.32 | 0.27 | 0.56 | 0.27 | 0.32 | 0.88 | 0.30 | 0.55 | 0.23 | 0.36 | 0.42 | 0.44 | 0.50 | TP (g/kg) |
| 22.26 | 17.06 | 14.34 | 22.65 | 18.37 | 16.42 | 30.46 | 23.43 | 24.88 | 24.80 | 16.65 | 18.16 | 24.09 | 16.11 | 21.23 | TK (g/kg) |
| 34.75 | 137.01 | 100.67 | 106.47 | 209.94 | 106.61 | 129.82 | 268.54 | 165.40 | 137.62 | 78.71 | 106.55 | 132.41 | 148.14 | 177.35 | AN (mg/kg) |
| 131.07 | 37.70 | 11.70 | 0.90 | 6.00 | 2.20 | 2.30 | 14.90 | 2.20 | 5.00 | 3.90 | 6.10 | 6.60 | 3.60 | 16.20 | AP (mg/kg) |
| 35.52 | 95.80 | 246.15 | 172.72 | 147.55 | 138.86 | 157.66 | 113.35 | 241.64 | 226.24 | 114.33 | 156.95 | 141.10 | 78.52 | 95.75 | AK (mg/kg) |
| 14.71 | 0.3037 | 0.3084 | 0.4342 | 0.3177 | 0.3140 | 0.2829 | 0.2602 | 0.2828 | 0.2888 | 0.2789 | 0.3310 | 0.2754 | 0.3560 | 0.2655 | Available boronm (g/kg) |
| 53.04 | 0.7559 | 0.7026 | 2.6049 | 6.6617 | 2.6990 | 2.6083 | 4.9162 | 3.1454 | 5.3284 | 2.3544 | 2.6302 | 2.5568 | 2.4957 | 4.3225 | Available copper (mg/kg) |
| 69.86 | 0.7909 | 1.0194 | 0.8840 | 5.0314 | 1.7027 | 6.4465 | 2.8695 | 1.4204 | 3.5210 | 0.8938 | 1.9485 | 2.1804 | 4.1079 | 1.7861 | Available zincm (g/kg) |
| 40.13 | 218.6641 | 146.9552 | 129.1538 | 131.7091 | 199.5054 | 161.9471 | 262.1424 | 101.9618 | 85.0319 | 100.1800 | 225.8220 | 92.7340 | 269.5029 | 109.3102 | Available iron (mg/kg) |
| 85.61 | 3.3612 | 10.2082 | 79.9308 | 9.4599 | 24.0042 | 22.0445 | 9.1684 | 31.2207 | 19.6051 | 10.9053 | 29.7598 | 17.2735 | 15.8877 | 21.4615 | Available manganese (mg/kg) |
| 10.62 | 0.1280 | 0.1151 | 0.0798 | 0.2687 | 0.1299 | 0.1417 | 0.1154 | 0.1305 | 0.1670 | 0.0539 | 0.3390 | 0.0994 | 0.1886 | 0.1808 | Available molybdenumm (g/kg) |
| 49.04 | 10.6523 | 8.2701 | 10.0684 | 25.1376 | 8.5963 | 6.7269 | 12.5154 | 15.0766 | 61.5709 | 7.4733 | 8.9735 | 22.2319 | 6.2195 | 26.1795 | Exchangeable calcium (Cmol/kg) |
| 51.18 | 2.1658 | 1.5937 | 2.7299 | 5.8062 | 1.9738 | 0.7644 | 2.5443 | 2.6297 | 3.2405 | 1.7516 | 1.5260 | 2.2487 | 1.5673 | 4.1401 | exchangeable magnesium (Cmol/kg) |
| 38.74 | 2.9719 | 2.1956 | 1.7021 | 3.4465 | 1.6712 | 1.6803 | 2.3427 | 1.7350 | 0.9839 | 1.0026 | 1.7218 | 1.0030 | 1.7832 | 1.4983 | Acid phosphatase (mg/g.24h） |
| 66.46 | 2.1749 | 1.1933 | 0.8065 | 2.1784 | 1.0181 | 0.3745 | 1.6329 | 0.7954 | 0.2190 | 0.7017 | 1.4087 | 0.1818 | 0.8045 | 0.3793 | Neutral phosphatase  (mg/g.24h) |
| 42.44 | 923.0452 | 689.4441 | 792.3858 | 1605.9757 | 856.6232 | 771.5827 | 1181.2450 | 1529.2111 | 2193.7693 | 880.7095 | 860.5219 | 1988.3386 | 718.1695 | 1588.1406 | Peroxidase (μmol/g.h) |
| 31.12 | 0.9416 | 0.6923 | 0.4039 | 0.8201 | 0.4446 | 0.5303 | 0.7849 | 0.5809 | 0.8716 | 0.3686 | 0.5278 | 0.4790 | 0.5666 | 0.4191 | Neutral protease (μmol/g.24h) |
| 68.97 | 0.1708 | 0.4549 | 0.6612 | 0.2239 | 0.6171 | 0.3990 | 0.3844 | 0.3982 | 1.3653 | 0.3144 | 0.6603 | 1.5602 | 0.4854 | 0.4620 | polyphenol oxidase (mg/g.24h) |
| 58.90 | 8.9104 | 7.7977 | 5.0584 | 23.0806 | 6.2177 | 7.8220 | 8.2193 | 9.7995 | 10.3577 | 3.9849 | 3.8295 | 4.9590 | 5.1542 | 9.1270 | Sucrase (mg/g.24h) |
| 161.41 | 0.0826 | 0.0673 | 0.0190 | 0.6306 | 0.0475 | 0.0042 | 0.0791 | 0.0934 | 0.2951 | 0.0109 | 0.0325 | 0.0004 | 0.0347 | 0.0619 | Urease (mg/g.24h) |
